# Walking forward or on hold: Could the ChatGPT be applied for seeking health information in neurosurgical settings?

**DOI:** 10.1002/ibra.12149

**Published:** 2024-03-09

**Authors:** Si‐Yu Yan, Yi‐Fan Liu, Lu Ma, Ling‐Long Xiao, Xin Hu, Rui Guo, Chao You, Rui Tian

**Affiliations:** ^1^ Department of Neurosurgery, West China Hospital Sichuan University Chengdu Sichuan China; ^2^ West China School of Medicine, West China Hospital Sichuan University Chengdu Sichuan China

**Keywords:** artificial intelligence, cerebrovascular disorders, health education

## Abstract

Self‐management is important for patients suffering from cerebrovascular events after neurosurgical procedures. An increasing number of artificial intelligence (AI)‐assisted tools have been used in postoperative health management. ChatGPT is a new trend dialog‐based chatbot that could be used as a supplemental tool for seeking health information. Responses from ChatGPT version 3.5 and 4.0 toward 13 questions raised by experienced neurosurgeons were evaluated in this exploratory study for their consistency and appropriateness blindly by the other three neurosurgeons. The readability of response text was investigated quantitively by word count and the Gunning Fog and Flesch–Kincaid indices. Results showed that the chatbot could provide relatively stable output between the two versions on consistency and appropriateness (*χ*² = 0.348). As for readability, there was a higher demand for readers to comprehend the output text in the 4.0 version (more counts of words; lower Flesch–Kincaid reading ease score; and higher Flesch–Kincaid grade level). In general, the capacity of ChatGPT to deliver effective health information is still under debate.

## Graphical abstract

Artificial intelligence (AI)‐assisted tools are becoming resources people seek for health information. The ChatGPT is a large language model (LLM) that has gone viral worldwide recently. As shown in the picture, which is similar to the human brain, the ChatGPT could give a direct response to complex queries based on complicated algorithms. It may have the potential to be used as a supplemental tool in self‐health management. The letter investigated the applications of the newly trending AI chatbot, ChatGPT, in neurosurgical settings.

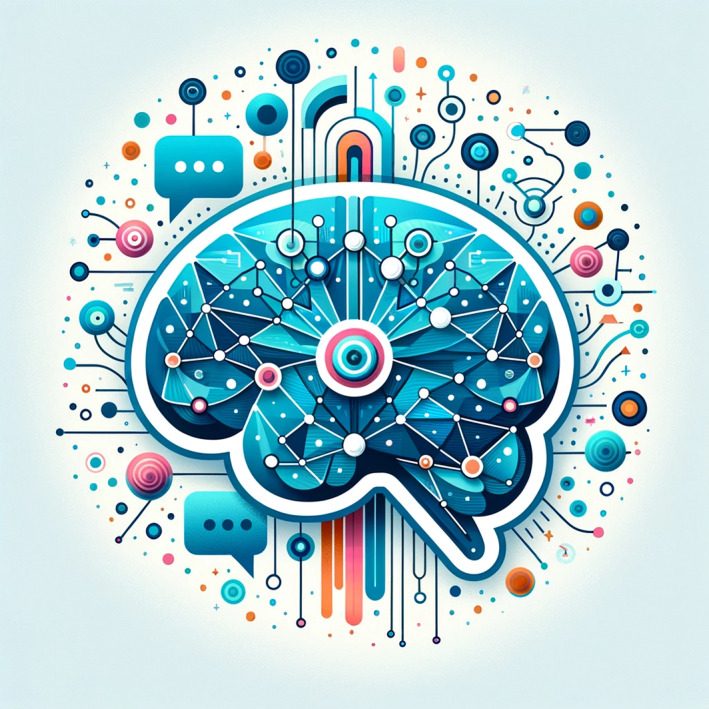

## 1 INTRODUCTION

Effective management of chronic illnesses (e.g., hypertension, stroke, etc.) requires patients' involvement in their healthcare beyond limited visits with health providers. In such a case, search engines, social media, and web‐based tools aided by artificial intelligence (AI) are becoming the main resources where people seek and share health information.^1^, ^2^ ChatGPT, trained with a method of Reinforcement Learning from Human Feedback (RLHF), is a new trend dialog‐based AI language model,^3^ in which a direct response to any complex queries from users could be generated within a short time. Therefore, this chatbot may have the potential to be used as a supplement tool in the management of chronic cases. The study is aimed at delivering a preliminary investigation, with a focus on cerebrovascular diseases in neurosurgical settings, to determine the utility of the ChatGPT for seeking health information.

## 2 METHODS

**Figure 1:**
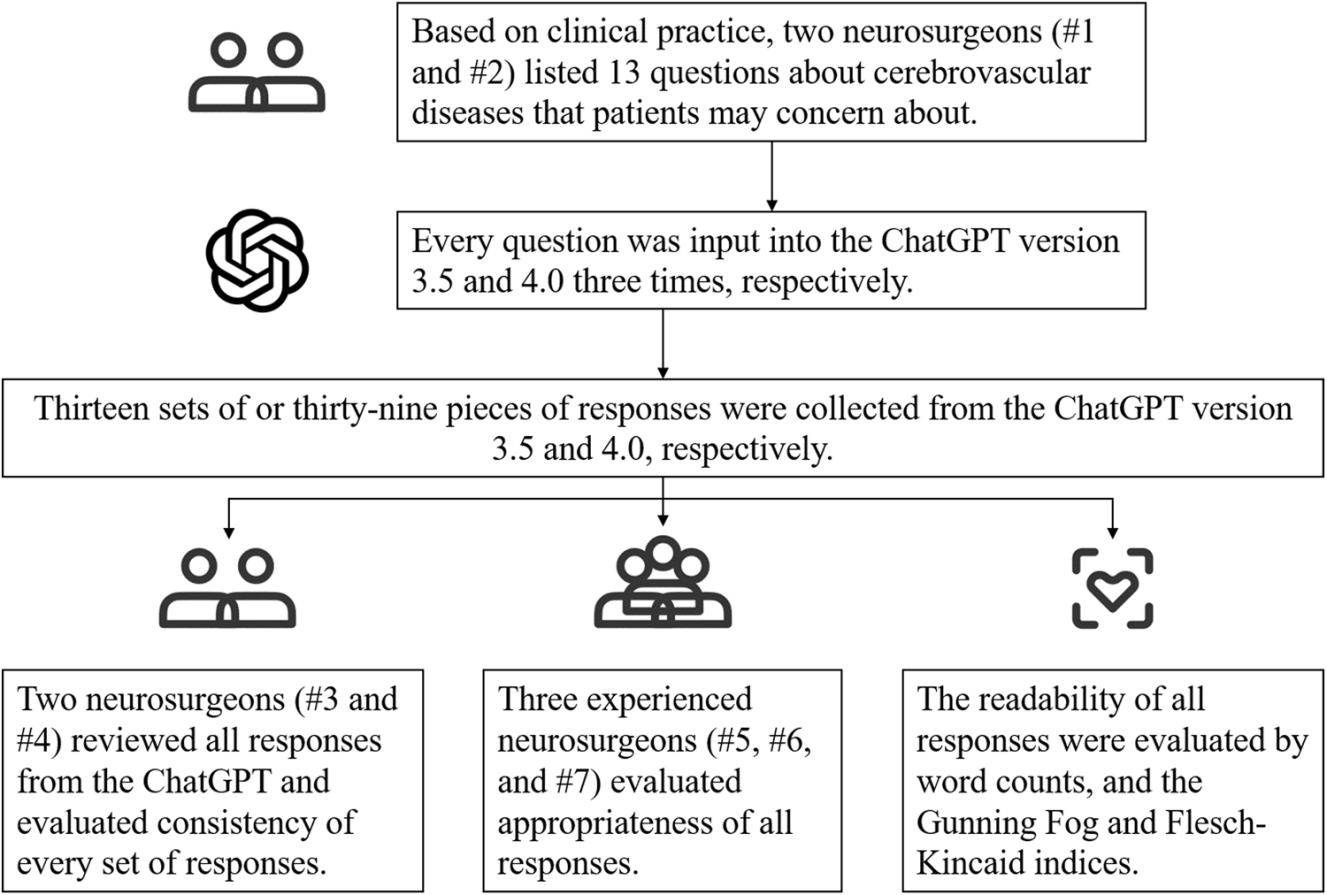
The workflow of accessing and evaluating responses from ChatGPT.

According to clinical experience, 13 questions (Table 1) about cerebrovascular events in neurosurgical settings that the general population may be concerned about were listed and input into the online ChatGPT 3.5 and 4.0 interface (https://chat.openai.com/), three times for each at a different time, respectively. All responses were recorded and reviewed by two independent reviewers (S.‐Y. Y., Y.‐F. L.) blindly for consistency of its key information (defined as the information that directly answers the questions) and the Supporting Information details (defined as the information of responses that further elucidate the key information to let readers understand the answers better). The definition of consistency was that, in response to the same questions, key information and Supporting Information details, respectively, were not mutually exclusive from each other. A set of responses was thought to be consistent only if both neurosurgeons agreed. Then, other three additional experienced neurosurgeons were assigned all sets of responses (L.‐L. X., X. H., R. G.) to grade them as appropriate (defined as all three responses were graded as appropriate) or inappropriate (defined as any of three responses was graded as inappropriate) blindly to judge if there was any information that may mislead readers. The Gunning Fog and Flesch–Kincaid indices (https://www.webfx.com/tools/read-able/) were used to evaluate the readability of the responses^4^ (Figure 1). The *χ* ^2^ test for consistency and appropriateness analysis and paired sample *t* test (*p* < 0.05, two‐tailed) for readability analysis were performed by the SPSS software (version 26, IBM Corp.) to compare the difference of quantitative measures between the ChatGPT 3.5 and 4.0.

**Table 1:** Evaluation of responses to 13 questions about cerebrovascular disease from the ChatGPT 3.5 and 4.0.

Question	Appropriateness		Word counts, means***		Flesch–Kincaid reading ease**		Flesch–Kincaid grade level*		Gunning Fog score	
	3.5^a^	4.0^b^	3.5	4.0	3.5	4.0	3.5	4.0	3.5	4.0
What is intracranial aneurysm?^c^	No	No	195.3	258.7	38.07	36.27	13.37	13.10	17.27	16.83
What is cerebral arteriosclerosis?	Yes	Yes	164.3	275.0	22.83	15.83	16.40	17.30	18.63	20.27
What is aspirin?	No	Yes	163.0	206.3	42.63	23.77	12.43	15.47	16.17	18.30
How to lower my blood pressure?	Yes	Yes	268.0	417.3	53.83	46.37	11.07	11.40	14.20	14.63
I have heart diseases. How to prevent cerebrovascular events?	Yes	Yes	274.3	404.3	41.87	42.47	13.27	12.07	16.17	15.37
How many daily steps do I need to prevent stroke?	Yes	Yes	173.3	247.7	38.80	31.17	13.77	14.87	18.60	17.80
What kind of exercise can I do after stroke?	Yes	Yes	224.7	334.7	30.97	26.03	14.33	15.27	19.30	19.30
Would hypertension lead to brain bleed?	No	Yes	142.3	285.7	48.70	42.43	11.83	12.33	15.17	15.37
I used to take aspirin, but I had a neurosurgical operation recently. When can I retreat aspirin?	Yes	Yes	161.7	150.3	30.93	29.33	14.53	14.57	18.97	18.73
Will I pass my intracranial aneurysm to my children?	No	Yes	147.7	206.3	22.20	15.30	16.03	17.00	20.97	21.97
What should I do if my blood pressure is still high after taking amlodipine?	Yes	No	190.7	255.7	38.30	37.10	13.13	13.37	16.80	16.60
Do my emotions have impact on my brain vessels?	No	No	178.7	330.3	40.97	31.60	13.43	14.63	18.30	18.30
I had a stroke. Can I drink coffee or tea?	No	Yes	199.7	284.7	45.10	37.23	13.17	13.67	17.37	17.37

^a^ ChatGPT version 3.5.

^b^ ChatGPT version 4.0.

^c^ Example response: “An intracranial aneurysm, also known as cerebral or brain aneurysm, is a bulging or ballooning in a weakened area of an artery in the brain. This weakened area of the artery is usually caused by a congenital defect or damage from injury or disease. Intracranial aneurysms are often asymptomatic and are discovered incidentally during imaging tests for other conditions. However, in some cases, they can cause symptoms such as headache, nausea, vomiting, neck pain, blurred or double vision, or even rupture, which can cause a life‐threatening condition called subarachnoid hemorrhage. Risk factors for intracranial aneurysm include smoking, high blood pressure, family history, and certain medical conditions such as polycystic kidney disease. Treatment options depend on the size, location, and severity of the aneurysm and may include observation, medications, or surgery.”

* *p* < 0.05

** *p* < 0.001

*** *p* < 0.0001.

## 3 RESULTS

Every set of three responses from ChatGPT 3.5 and 4.0 regarding 13 listed questions (Supporting Information) had good consistency of key information (ChatGPT 3.5 vs. 4.0, 39/39 [100%] vs. 39/39 [100%]); however, the Supporting Information details of every three answers were not in accordance with each other in most cases (10/13 [77.0%] vs. 11/13 [84.6%]). 7/13 [53.9%] and 10/13 [77.0%] in sets (*p* = 0.411), or 31/39 [79.5%] and 33/39 [84.6%] in the individual piece (*χ*² = 0.348, *p* = 0.555) of responses from ChatGPT 3.5 and 4.0 were graded as appropriate, respectively (Table 1). Most of the responses had warning messages if the chatbot thinks readers in the face of settings needed health professionals to intervene.

Compared to ChatGPT 3.5, responses from ChatGPT 4.0 had more counts of words (mean difference, 90.3 [95% confidence interval, CI, 61–119]; *p* < 0.0001), lower Flesch–Kincaid reading ease score (mean difference, −6.18 [95% CI, −9.15 to −3.19], *p* < 0.001, a lower score indicates more difficulties in reading), and higher Flesch–Kincaid grade level (mean difference, 0.64 [95% CI, 0.48–1.23], *p* = 0.036, a higher grade level indicates more difficulties in reading). There was no difference in the Gunning Fog score (mean difference, 0.36 [95% CI, −0.18 to 0.91], *p* = 0.176, a lower score indicates greater ease of reading).

## 4 DISCUSSION

Different from other attempts,^5^, ^6^ this study reveals that the capacity of this new trend AI interactive model to deliver effective health information should be still under debate. Although most of the responses contained cautious information and the key information of every set of three responses was consistent and appropriate for the general population, there is still a certain risk of misleading readers due to the ways of expression and inaccurate Supporting Information details. In most cases, the Supporting Information details of the same question were different (10/13 [77.0%] vs. 11/13 [84.6%]). In this way, the output patients received in ChatGPT may not be consistent. Unlike traditional search engines, which may struggle with ambiguous medical terminology and inundate users with redundant information, ChatGPT can be harnessed effectively to enhance the long‐term management of cerebrovascular diseases and other chronic conditions. It serves as a valuable resource for individuals seeking basic medical advice, particularly in noncritical situations such as compliance and rehabilitation. Besides, the chatbot has the potential to improve the doctor–patient relationship and patient education in neurosurgical clinical practice by decreasing communication costs with this complementary health information resource. But at the same time, there are raising concerns about the reliability, effectiveness, abuse, and so on, problems of the chatbot,^7^ and the model is not designed for medical purposes only. Therefore, further investigation and supervision should be brought to the forefront among the developers, the health‐related professions, and the users.

The iteration speed of AI technology tools is fast, an updated version of ChatGPT may be a way to remove the concerns and provide a more accurate response regarding the questions input into the chatbot. Indeed, results showed that the new version had a higher rate of accuracy, which is following its development trajectories.^8^ Nevertheless, the output of the context by the upgraded chatbot had a higher demand for readers to comprehend the messages they receive (more counts of words, *p* < 0.0001; lower Flesch–Kincaid reading ease score, *p* < 0.001; and higher Flesch–Kincaid grade level, *p* = 0.036), which may undermine its advantages and confuse the readers with complex texts. The reasons could be attributed to the new version chatbot trained with updated information as well as algorithms. Nevertheless, the users could receive a more concise response by asking the chatbot repeatedly.

This study has some limitations. The chatbot was trained using RLHF to reduce harmful outputs, but the responses still lack credible sources and evidence. The reliability and consistency of answers outside the questions covered in this study cannot be guaranteed, and it may provide misleading information for specific populations. The hysteretic nature of data used for training the AI model also prevents it from offering the latest medical information to users.^3^ Besides, this exploratory study assessed the AI model mainly from consistency, appropriateness, and readability; however, a more comprehensive and technical way should be adopted, to conduct the investigation within a large‐scale user population instead of cerebrovascular‐related professionals with subjective preference and potential bias. Further studies are needed to compare the performance of this AI model with other AI tools and conventional medical advice sources in a quantitative manner.

## AUTHOR CONTRIBUTIONS

Rui Tian had full access to all data in the study and took responsibility for the integrity of the data and the accuracy of the data analysis. Si‐Yu Yan designed the study, acquired data, reviewed for consistency of data, analyzed data, and drafted the manuscript. Yi‐Fan Liu designed the study, acquired data, reviewed for consistency of data, analyzed data, and revised the manuscript. Ling‐Long Xiao reviewed for appropriateness of data. Xin Hu reviewed for appropriateness of data. Rui Guo reviewed for appropriateness of data. Lu Ma supervised the study, and revised the manuscript. Chao You supervised the study. Rui Tian designed the study, supervised the study, and revised the manuscript.

## CONFLICT OF INTEREST STATEMENT

The authors declare no conflict of interest.

## ETHICS STATEMENT

The authors have nothing to report.

## Supporting information

Supporting information.

## Data Availability

The data sets used and/or analyzed during the current study are available from the corresponding author on reasonable request.

## References

[1] Miller T , Stockley R , Drummond A , et al. Online advice for the symptomatic management of post‐stroke fatigue: a scoping review. J Psychosom Res. 2022;162:111039. 10.1016/j.jpsychores.2022.111039 36179422

[2] Meyer AND , Giardina TD , Spitzmueller C , Shahid U , Scott TMT , Singh H . Patient perspectives on the usefulness of an artificial Intelligence‐assisted symptom checker: cross‐sectional survey study. J Med Internet Res. 2020;22(1):e14679. 10.2196/14679 32012052 PMC7055765

[3] ChatGPT: optimizing language models for dialogue. OpenAI; 2022. Accessed December 1, 2022. https://openai.com/blog/chatgpt/

[4] Terblanche M . Examining the readability of patient‐informed consent forms. Open Access J Clin Trials. 2010;2:157‐162. 10.2147/OAJCT.S13608

[5] Sarraju A , Bruemmer D , Van Iterson E , Cho L , Rodriguez F , Laffin L . Appropriateness of cardiovascular disease prevention recommendations obtained from a popular online chat‐based artificial intelligence model. JAMA. 2023;329(10):842‐844. 10.1001/jama.2023.1044 36735264 PMC10015303

[6] Johnson SB , King AJ , Warner EL , Aneja S , Kann BH , Bylund CL . Using ChatGPT to evaluate cancer myths and misconceptions: artificial intelligence and cancer information. JNCI Cancer Spectr. 2023;7(2):pkad015. 10.1093/jncics/pkad015 36929393 PMC10020140

[7] Stokel‐Walker C . ChatGPT listed as author on research papers: many scientists disapprove. Nature. 2023;613(7945):620‐621. 36653617 10.1038/d41586-023-00107-z

[8] GPT‐4 is OpenAI's most advanced system, producing safer and more useful responses . OpenAI; 2023. Accessed March 5, 2024. https://openai.com/product/gpt-4

