# Supplementary material for: Walking forward or on hold: Could the ChatGPT be applied for seeking health information in neurosurgical settings?
Source: Ibrain. 2024 Mar 9;10(1):111–5. doi: 10.1002/ibra.12149 (PMC11045188; doi:10.1002/ibra.12149)
Supplement: Supplementary file 1 — Supporting information. [file IBRA-10-111-s001.docx]

Supplement materials The questions list and responses from the ChatGPT 3.5 and 4.0

| Question 1 | | What is intracranial aneurysm? |
| --- | --- | --- |
|  |  | An intracranial aneurysm, also known as cerebral or brain aneurysm, is a bulging or ballooning in a weakened area of an artery in the brain. This weakened area of the artery is usually caused by a congenital defect or damage from injury or disease. |
|  | Re 1 | Intracranial aneurysms are often asymptomatic and are discovered incidentally during imaging tests for other conditions. However, in some cases, they can cause symptoms such as headache, nausea and vomiting, neck pain, blurred or double vision, or even rupture, which can cause a life-threatening condition called subarachnoid hemorrhage (SAH). |
|  |  | Risk factors for intracranial aneurysm include smoking, high blood pressure, family history, and certain medical conditions such as polycystic kidney disease. Treatment options depend on the size, location, and severity of the aneurysm and may include observation, medications, or surgery. |
|  |  | An intracranial aneurysm is a bulging or ballooning of a blood vessel in the brain. This can occur when the wall of a blood vessel becomes  weakened or damaged, causing it to stretch and form a sac-like bulge. |
| ChatGPT 3.5 |  | Intracranial aneurysms can occur in any part of the brain, but are most commonly found in the arteries at the base of the brain, known as the circle of Willis. They can vary in size and shape, and may be small and asymptomatic, or larger and cause symptoms such as headache, blurred vision, or neurological deficits. |
|  | Re 2 | If an intracranial aneurysm ruptures or bursts, it can cause a type of stroke called a subarachnoid hemorrhage, which can be life- threatening. Symptoms of a ruptured aneurysm may include sudden, severe headache, nausea and vomiting, neck stiffness, sensitivity to light, and loss of consciousness. |
|  |  | Risk factors for intracranial aneurysms include high blood pressure, smoking, family history of the condition, and certain medical conditions such as connective tissue disorders. They can be diagnosed using imaging tests such as MRI, CT scan, or angiogram. |
|  |  | Treatment for intracranial aneurysms depends on the size and location of the aneurysm, as well as the individual's overall health and other factors. Options may include observation, surgical  clipping, or endovascular coiling, which involves inserting a catheter |

|  |  | through an artery in the groin and guiding it to the site of the aneurysm to fill it with coils or a stent to prevent further bulging or  rupture. |
| --- | --- | --- |
|  | Re 3 | An intracranial aneurysm is a bulging, weakened area in the wall of a blood vessel in the brain. The bulging area can fill with blood and put pressure on surrounding tissues or nerves. If the aneurysm ruptures, it can cause bleeding in the brain, which can be life- threatening.  Intracranial aneurysms are relatively common, with an estimated 2- 3% of people having an unruptured aneurysm in their brain. They are most often found in people between the ages of 35 and 60, and are more common in women than in men.  Most intracranial aneurysms do not cause symptoms and are discovered incidentally during brain imaging for another condition. However, in some cases, aneurysms can cause symptoms such as headache, blurred vision, and changes in speech or balance.  The risk of an aneurysm rupturing depends on its size, location, and other factors. Small aneurysms are less likely to rupture than larger ones. Treatment options for aneurysms depend on several factors, including the size and location of the aneurysm, the risk of rupture, and the individual's overall health and medical history. Treatment options may include monitoring the aneurysm, surgery to clip or remove the aneurysm, or endovascular coiling to fill the aneurysm  with a special material to prevent it from rupturing. |
| ChatGPT 4.0 | Re 1 | An intracranial aneurysm, also known as a cerebral or brain aneurysm, is a weak or abnormal bulging spot on the wall of a blood vessel in the brain. This bulge is caused by the weakening of the vessel wall, which can be due to various factors, such as congenital defects, atherosclerosis, or head injuries. Over time, the pressure of blood flow within the artery can cause the weakened area to enlarge and form a balloon-like protrusion.  Intracranial aneurysms are typically classified into two types: saccular and fusiform. Saccular aneurysms, also known as berry aneurysms, are the most common type, accounting for about 90% of cases. They have a rounded shape and usually arise at the branching points of arteries. Fusiform aneurysms, on the other hand, are less common and involve a more uniform, elongated dilation of the blood vessel wall.  Many intracranial aneurysms remain asymptomatic and are |

|  |  | discovered incidentally during imaging studies for other reasons. However, if an aneurysm ruptures, it can lead to a subarachnoid hemorrhage, a life-threatening type of stroke caused by bleeding into the space surrounding the brain. This can result in severe headaches, loss of consciousness, seizures, and other neurological symptoms.  Treatment for intracranial aneurysms depends on the size, location, and risk of rupture. In some cases, particularly for small, unruptured aneurysms, a "watchful waiting" approach with regular monitoring might be recommended. For larger or high-risk aneurysms, surgical or endovascular interventions, such as clipping or coiling, may be  necessary to prevent rupture and reduce the risk of complications. |
| --- | --- | --- |
|  | Re 2 | An intracranial aneurysm, also known as a brain aneurysm, is a bulging or weakened area in the wall of an artery in the brain. The bulging occurs due to the pressure of blood flowing through the artery, which causes the weakened wall to balloon outward. Intracranial aneurysms can vary in size, and some may remain small and stable without causing any problems. However, larger aneurysms or those that continue to grow can pose a serious risk.  If an intracranial aneurysm ruptures, it can lead to a subarachnoid hemorrhage, which is a type of stroke caused by bleeding into the space surrounding the brain. A ruptured aneurysm can be life- threatening and requires immediate medical attention. Symptoms of a ruptured aneurysm can include a sudden, severe headache, nausea, vomiting, a stiff neck, blurred or double vision, sensitivity to light, seizures, or loss of consciousness.  Risk factors for developing an intracranial aneurysm include age, family history, smoking, hypertension (high blood pressure), and certain genetic conditions. Treatment for an unruptured aneurysm depends on its size, location, and the patient's overall health. Options may include close monitoring, medications to manage blood pressure, or surgical interventions such as endovascular coiling or  surgical clipping. |
|  | Re 3 | An intracranial aneurysm, also known as a cerebral aneurysm or brain aneurysm, is a weakened or bulging area in the wall of a blood vessel in the brain. The bulge occurs because the blood vessel wall is weakened or damaged, which allows blood pressure to cause a localized dilation or ballooning of the vessel. Intracranial aneurysms can vary in size, shape, and location within the brain's blood vessels.  There are different types of intracranial aneurysms, with the most common types being: |

|  |  | Saccular aneurysms (berry aneurysms): These are round, sac-like bulges that occur at a branching point of a cerebral artery. Saccular aneurysms are the most common type of intracranial aneurysm and are more likely to rupture compared to other types.  Fusiform aneurysms: These aneurysms cause the entire circumference of the blood vessel to bulge outwards, creating a more uniform, spindle-shaped dilation. Fusiform aneurysms are less likely to rupture than saccular aneurysms.  Many intracranial aneurysms do not cause symptoms and are discovered incidentally during brain imaging for other reasons. However, if an aneurysm grows large, it can press on nearby brain tissue and nerves, causing symptoms such as headaches, vision problems, or facial pain. A ruptured intracranial aneurysm is a medical emergency, as it can lead to bleeding within the brain (hemorrhagic stroke), which can cause severe neurological damage or even death.  Risk factors for developing intracranial aneurysms include: Age: The risk of developing an aneurysm increases with age.  Family history: A family history of intracranial aneurysms increases the risk.  Genetic conditions: Some genetic conditions, such as polycystic kidney disease, Marfan syndrome, or Ehlers-Danlos syndrome, can increase the risk of aneurysms.  Smoking: Smoking damages blood vessels and increases the risk of aneurysms.  Hypertension: High blood pressure can contribute to the development and growth of aneurysms.  If you are concerned about intracranial aneurysms or are experiencing symptoms, it is essential to consult with a healthcare  professional for a proper evaluation and diagnosis. |
| --- | --- | --- |

| Question 2 | | What is cerebral arteriosclerosis? |
| --- | --- | --- |
| ChatGPT 3.5 | Re 1 | Cerebral arteriosclerosis, also known as cerebral atherosclerosis, is a condition in which the arteries that supply blood to the brain become hardened and narrowed due to the buildup of plaque, a combination of cholesterol, fat, calcium, and other substances.  As the arteries become narrower, blood flow to the brain is reduced, which can lead to a range of symptoms including headaches, dizziness, confusion, difficulty concentrating, memory loss, and mood changes. In severe cases, cerebral arteriosclerosis can cause a stroke or transient ischemic attack (TIA), also known as a "mini- stroke."  Cerebral arteriosclerosis is often associated with aging and other risk factors such as high blood pressure, smoking, diabetes, high cholesterol, and a family history of the condition. Treatment options may include lifestyle changes such as a healthy diet and regular exercise, medications to control blood pressure and cholesterol, and  in some cases, surgical procedures to open or bypass blocked arteries. |
|  | Re 2 | Cerebral arteriosclerosis, also known as cerebral atherosclerosis, is a condition characterized by the hardening and narrowing of the arteries that supply blood to the brain. This condition is caused by the buildup of plaque (fatty deposits) inside the walls of the arteries, which leads to reduced blood flow to the brain.  The symptoms of cerebral arteriosclerosis can include headaches, dizziness, confusion, difficulty speaking or understanding language, memory problems, and changes in behavior or mood. In severe cases, cerebral arteriosclerosis can cause a stroke, which can lead to long- term disability or even death.  Risk factors for cerebral arteriosclerosis include high blood pressure, high cholesterol, smoking, diabetes, obesity, and a family history of heart disease or stroke. Treatment for cerebral arteriosclerosis may include medications to lower blood pressure and cholesterol, lifestyle changes such as diet and exercise, and in some cases, surgery to  remove plaque from the arteries or to bypass blocked arteries. |
|  | Re 3 | Cerebral arteriosclerosis, also known as cerebral atherosclerosis or intracranial atherosclerosis, is a condition in which the walls of arteries in the brain become thickened and hardened due to the buildup of fatty deposits called plaques. The condition can restrict blood flow to the brain, leading to a range of neurological symptoms, including stroke. |

|  |  | Arteriosclerosis is a general term for the thickening and hardening of arteries due to the accumulation of plaque. When this occurs in the arteries of the brain, it is called cerebral arteriosclerosis. The condition is typically caused by risk factors such as high blood pressure, high cholesterol, smoking, and diabetes.  Symptoms of cerebral arteriosclerosis can include headache, dizziness, confusion, memory loss, and difficulty with movement or speech. In some cases, cerebral arteriosclerosis can lead to stroke, which occurs when blood flow to part of the brain is blocked or when a blood vessel in the brain ruptures.  Treatment for cerebral arteriosclerosis typically focuses on managing underlying risk factors such as high blood pressure and high cholesterol through lifestyle changes, medications, and other interventions. In some cases, surgery may be necessary to remove or  bypass a blocked artery. |
| --- | --- | --- |
| ChatGPT 4.0 | Re 1 | Cerebral arteriosclerosis, also known as cerebrovascular atherosclerosis or simply cerebrovascular disease, is a condition where the arteries supplying blood to the brain become narrowed, hardened, or less elastic due to the buildup of fatty deposits, cholesterol, and other substances on the inner walls of the arteries. This process is called atherosclerosis, and when it occurs in the arteries of the brain, it's specifically referred to as cerebral arteriosclerosis.  The buildup of plaque in the arteries can reduce blood flow to the brain, and in some cases, the plaque can rupture, causing a blood clot to form. Depending on the location and severity of the blockage, cerebral arteriosclerosis can lead to various complications, including:  Transient ischemic attack (TIA): Also known as a mini-stroke, TIA is a temporary disruption of blood flow to the brain that lasts for a short period, usually less than 24 hours. TIAs often serve as a warning sign for a potential future stroke.  Ischemic stroke: This is the most common type of stroke and occurs when an artery supplying blood to the brain is blocked, either by a blood clot or atherosclerotic plaque, leading to a lack of oxygen and nutrients in the affected area of the brain.  Hemorrhagic stroke: This type of stroke happens when a weakened blood vessel ruptures and bleeds into the brain tissue. Cerebral arteriosclerosis can contribute to the weakening of blood vessels, |

|  |  | increasing the risk of hemorrhage. |
| --- | --- | --- |
|  |  | Vascular dementia: Reduced blood flow to the brain due to cerebral arteriosclerosis can also lead to cognitive decline, memory loss, and other symptoms of dementia. |
|  |  | Risk factors for cerebral arteriosclerosis include aging, high blood pressure, high cholesterol, smoking, obesity, diabetes, and a family history of heart or vascular disease. Preventive measures, such as maintaining a healthy lifestyle, regular exercise, eating a balanced diet, and managing risk factors, can help reduce the risk of developing this condition. Treatment options include medications to control blood pressure and cholesterol levels, as well as lifestyle modifications and, in some cases, surgical interventions to remove or  bypass the blockage. |
|  |  | Cerebral arteriosclerosis is a condition where the blood vessels that supply the brain (cerebral arteries) become narrowed or hardened due to the accumulation of plaque, which is primarily composed of fat, cholesterol, calcium, and other substances. This build-up can impede blood flow to the brain, potentially leading to a range of neurological issues. |
|  |  | Cerebral arteriosclerosis is a form of atherosclerosis, a generalized condition affecting arteries throughout the body. It is particularly concerning when it affects the cerebral arteries because the brain is highly sensitive to reductions in blood flow, and the resulting oxygen and nutrient deficiency can lead to serious complications. |
|  |  | Risk factors for cerebral arteriosclerosis include: |
|  | Re 2 |  |
|  |  | Age  High blood pressure (hypertension) High cholesterol levels  Diabetes Obesity Smoking  Family history of heart disease or stroke Sedentary lifestyle  Excessive alcohol consumption Chronic inflammation  Cerebral arteriosclerosis can lead to various neurological complications, such as transient ischemic attacks (TIAs), ischemic strokes, and cognitive decline or dementia. Early diagnosis and  management of the condition and its risk factors are essential for |

|  |  | reducing the likelihood of these complications. Treatment options may include lifestyle changes, medication to manage blood pressure and cholesterol, and, in some cases, surgical interventions to restore  blood flow. |
| --- | --- | --- |
|  | Re 3 | Cerebral arteriosclerosis, also known as cerebrovascular atherosclerosis, is a condition in which the arteries in the brain become narrowed and hardened due to the buildup of fatty deposits, cholesterol, and other substances. This buildup, called plaque, can reduce blood flow to the brain, potentially leading to stroke or other cerebrovascular events.  Cerebral arteriosclerosis is a type of atherosclerosis, a more general term referring to the hardening and narrowing of arteries throughout the body. Atherosclerosis can affect arteries in various parts of the body, including the heart (coronary artery disease), legs (peripheral artery disease), and kidneys (renal artery disease).  Risk factors for cerebral arteriosclerosis include:  Age: The risk of developing arteriosclerosis increases with age. High blood pressure: Hypertension can damage the walls of the arteries, making them more susceptible to plaque buildup.  High cholesterol: Elevated levels of low-density lipoprotein (LDL) cholesterol can contribute to plaque formation.  Smoking: Smoking damages blood vessels and contributes to the development of atherosclerosis.  Diabetes: High blood sugar levels can damage blood vessels and increase the risk of atherosclerosis.  Obesity: Excess body weight can contribute to high blood pressure, high cholesterol, and inflammation, all of which are risk factors for atherosclerosis.  Family history: A family history of atherosclerosis or other cardiovascular diseases can increase your risk.  Managing the risk factors for cerebral arteriosclerosis is crucial to prevent complications such as stroke. Lifestyle changes, including a healthy diet, regular exercise, smoking cessation, and maintaining a healthy weight, can help reduce the risk of atherosclerosis. In some cases, medications may be prescribed to manage high blood pressure, high cholesterol, or other related conditions.  If you are concerned about cerebral arteriosclerosis or your risk  factors, consult with a healthcare professional for personalized advice and appropriate management strategies. |

| Question 3 | | What is aspirin? |
| --- | --- | --- |
| ChatGPT 3.5 | Re 1 | Aspirin is a commonly used over-the-counter medication that belongs to a group of drugs called nonsteroidal anti-inflammatory drugs (NSAIDs). It is used to relieve pain, reduce inflammation, and lower fever.  Aspirin works by blocking the production of prostaglandins, which are chemicals that cause pain, inflammation, and fever. As a result, aspirin can be effective in relieving headaches, toothaches, menstrual cramps, and other types of pain. It can also help reduce inflammation in conditions such as arthritis.  In addition to its pain-relieving properties, aspirin is also a blood- thinning medication. It can help prevent blood clots from forming and reduce the risk of heart attacks and strokes in people who have a history of cardiovascular disease.  Aspirin should be used as directed, and it is important to follow the recommended dosage and frequency to avoid potential side effects such as stomach upset, bleeding, or allergic reactions. As with any medication, it is important to speak with a healthcare provider before  starting or stopping aspirin therapy. |
|  | Re 2 | Aspirin is a commonly used medication that belongs to a class of drugs called nonsteroidal anti-inflammatory drugs (NSAIDs). It is used primarily for pain relief, reduction of fever, and anti- inflammatory effects.  Aspirin works by inhibiting the production of certain enzymes called cyclooxygenases (COX-1 and COX-2), which are involved in the production of prostaglandins, hormone-like substances that cause inflammation, pain, and fever. By blocking the production of these enzymes, aspirin can reduce inflammation and pain and lower fever.  In addition to its pain-relieving and anti-inflammatory effects, aspirin is also used for its blood-thinning properties. It can help prevent blood clots from forming, which can reduce the risk of heart attack or stroke in people who have cardiovascular disease or who are at high risk for these conditions.  Aspirin is available over-the-counter in various doses, and it is also available by prescription in higher doses. It is generally safe and effective when used as directed, but it can cause side effects such as stomach upset, bleeding, and allergic reactions in some people. It is  important to talk to a healthcare provider before taking aspirin, |

|  |  | especially if you have any medical conditions or are taking other  medications. |
| --- | --- | --- |
|  | Re 3 | Aspirin, also known as acetylsalicylic acid, is a medication that belongs to a class of drugs called nonsteroidal anti-inflammatory drugs (NSAIDs). Aspirin is commonly used to relieve pain, reduce inflammation, and lower fever.  In addition to its use as an over-the-counter pain reliever, aspirin is also prescribed by doctors to prevent blood clots from forming. This is because aspirin is a blood thinner that can reduce the risk of heart attack and stroke by preventing blood platelets from clumping together and forming clots.  Aspirin is also sometimes used in low doses to prevent certain types of cancer, particularly colon cancer, and to reduce the risk of recurrent pre-eclampsia in pregnant women.  Like all medications, aspirin can cause side effects, and it may not be suitable for everyone. Common side effects of aspirin include stomach upset, nausea, and heartburn. In some people, aspirin can cause more serious side effects such as bleeding in the stomach or intestines, allergic reactions, and worsening of asthma symptoms. Aspirin should be used with caution in people with certain medical conditions, including kidney disease, liver disease, and bleeding  disorders. |
| ChatGPT 4.0 | Re 1 | Aspirin, also known by its chemical name acetylsalicylic acid (ASA), is a widely used medication with analgesic (pain-relieving), antipyretic (fever-reducing), and anti-inflammatory properties. It belongs to a class of drugs called nonsteroidal anti-inflammatory drugs (NSAIDs) and has been in use for over a century as a common over-the-counter medication for various ailments.  Aspirin works by inhibiting the production of prostaglandins, which are hormone-like substances that play a role in pain, inflammation, and fever. Prostaglandins are synthesized by the enzyme cyclooxygenase (COX). Aspirin inhibits COX, thereby reducing the production of prostaglandins and alleviating symptoms.  In addition to its analgesic, antipyretic, and anti-inflammatory effects, aspirin is also used for its antiplatelet properties. In low doses, aspirin can help prevent blood clots by inhibiting platelet aggregation, which is the clumping together of platelets in the blood. Due to this effect, low-dose aspirin is often prescribed as a preventive  measure for individuals at risk of heart attacks, strokes, and other |

|  |  | cardiovascular events. |
| --- | --- | --- |
|  |  | However, aspirin is not without side effects. It can cause gastrointestinal upset, including stomach pain, heartburn, and nausea. In some cases, it may also lead to more severe gastrointestinal issues, such as ulcers and bleeding. Moreover, aspirin can interact with other medications and is not suitable for everyone. For instance, it is generally not recommended for children and teenagers with fever, as it has been associated with the development of a rare but potentially life-threatening condition called Reye's syndrome. |
|  |  | It is essential to consult a healthcare professional before starting or stopping aspirin therapy, especially when using it for preventive  purposes or in combination with other medications. |
|  |  | Aspirin, also known as acetylsalicylic acid (ASA), is a widely used medication that belongs to a group of drugs called nonsteroidal anti- inflammatory drugs (NSAIDs). It is derived from salicylic acid, a compound originally found in the bark of the willow tree. Aspirin has several therapeutic effects, including: |
|  |  | Analgesic effect: Aspirin provides pain relief by reducing the production of prostaglandins, which are chemicals responsible for causing pain and inflammation. |
|  |  | Antipyretic effect: Aspirin helps reduce fever by acting on the hypothalamus, the part of the brain responsible for regulating body temperature. |
|  | Re 2 | Anti-inflammatory effect: Aspirin inhibits the production of prostaglandins, which are responsible for inflammation, thus helping to reduce swelling and discomfort. |
|  |  | Antiplatelet effect: Aspirin prevents blood clots by inhibiting the action of platelets, which play a crucial role in clot formation. This effect is especially useful for reducing the risk of heart attack and stroke in certain individuals. |
|  |  | Aspirin is available over-the-counter and is commonly used to treat mild to moderate pain, such as headaches, toothaches, and muscle aches. It can also be prescribed in low doses to reduce the risk of heart attack and stroke in individuals with specific risk factors. However, aspirin is not recommended for everyone, as it can cause  side effects and interact with other medications. It is essential to |

|  |  | consult a healthcare professional before starting aspirin therapy. |
| --- | --- | --- |
|  | Re 3 | Aspirin, also known as acetylsalicylic acid (ASA), is a widely used over-the-counter medication that belongs to a group of drugs called nonsteroidal anti-inflammatory drugs (NSAIDs). It is commonly used to relieve pain, reduce inflammation, and lower fever. Additionally, aspirin has an antiplatelet effect, meaning it can prevent blood platelets from sticking together and forming clots.  Due to its antiplatelet properties, aspirin is often prescribed in low doses (also known as baby aspirin or low-dose aspirin) for people at risk of heart attack, stroke, or other cardiovascular events. By inhibiting the formation of blood clots, aspirin can help reduce the risk of blockages in the arteries that supply blood to the heart and brain.  It is important to note that aspirin is not appropriate for everyone, and its use may be associated with certain risks and side effects, such as gastrointestinal bleeding, allergic reactions, or interactions with other medications. Therefore, it is essential to consult with a healthcare professional before starting or stopping aspirin therapy, particularly if you have a history of gastrointestinal issues, bleeding disorders, or are taking other medications.  In summary, aspirin is a widely used medication with analgesic, anti- inflammatory, antipyretic, and antiplatelet properties. It is often used to relieve pain, reduce fever, and prevent blood clots, but its use should be guided by a healthcare professional to ensure safety and  efficacy. |

| Question 4 | | How to lower my blood pressure? |
| --- | --- | --- |
| ChatGPT 3.5 | Re 1 | There are several lifestyle changes that can help lower blood pressure:  Maintain a healthy weight: Being overweight or obese can increase your risk of high blood pressure, so maintaining a healthy weight can help lower your blood pressure.  Exercise regularly: Regular physical activity, such as walking, swimming, or cycling, can help lower blood pressure. Aim for at least 30 minutes of moderate-intensity exercise most days of the week.  Eat a healthy diet: A diet that is rich in fruits, vegetables, whole grains, lean protein, and low-fat dairy products can help lower blood pressure. Limit your intake of saturated and trans fats, salt, and added sugars.  Reduce sodium intake: Sodium can raise blood pressure, so it is important to limit your intake of salt and salty foods. Aim for less than 2,300 milligrams of sodium per day, and even less if you have high blood pressure.  Limit alcohol consumption: Drinking too much alcohol can raise blood pressure, so it is important to limit your alcohol intake. For men, limit consumption to no more than two drinks per day, and for women, limit to no more than one drink per day.  Manage stress: Chronic stress can contribute to high blood pressure, so it is important to find ways to manage stress. This may include relaxation techniques such as meditation, yoga, or deep breathing exercises.  In addition to lifestyle changes, medications may also be prescribed to help lower blood pressure. It is important to work with a healthcare provider to develop a plan that is tailored to your individual needs. |
|  | Re 2 | There are several lifestyle changes you can make to help lower your blood pressure naturally. Here are some tips:  Maintain a healthy weight: Being overweight or obese can increase your risk of high blood pressure. Losing weight through a healthy diet and regular exercise can help lower your blood pressure.  Exercise regularly: Regular physical activity can help lower your blood pressure. Aim for at least 150 minutes of moderate-intensity |

|  |  | exercise or 75 minutes of vigorous-intensity exercise per week.  Eat a healthy diet: Eating a diet rich in fruits, vegetables, whole grains, and lean protein sources, and low in saturated and trans fats, cholesterol, and sodium can help lower your blood pressure.  Reduce sodium intake: Consuming too much salt can increase your blood pressure. Aim to consume less than 2,300 milligrams of sodium per day.  Limit alcohol intake: Drinking too much alcohol can increase your blood pressure. Men should limit their intake to no more than two drinks per day, and women should limit their intake to no more than one drink per day.  Quit smoking: Smoking can increase your blood pressure and damage your blood vessels. Quitting smoking can help lower your blood pressure and reduce your risk of heart disease.  Manage stress: Chronic stress can contribute to high blood pressure. Find ways to manage stress, such as practicing relaxation techniques, getting enough sleep, and engaging in enjoyable activities.  In addition to these lifestyle changes, your healthcare provider may recommend medications to help lower your blood pressure if necessary. It is important to talk to your healthcare provider before making any significant changes to your lifestyle or starting any new  medications. |
| --- | --- | --- |
|  | Re 3 | There are several lifestyle changes and medications that can help lower blood pressure:  Maintain a healthy weight: Being overweight or obese can increase your blood pressure, so maintaining a healthy weight can help to lower it.  Follow a healthy diet: A diet that is high in fruits, vegetables, whole grains, and low-fat dairy products, and low in saturated and trans fats can help to lower blood pressure. It is also recommended to limit salt intake to less than 2,300 milligrams per day (or even less if advised by a doctor).  Exercise regularly: Regular physical activity, such as brisk walking, swimming, cycling, or other aerobic exercises, can help to lower blood pressure. |

|  |  | Limit alcohol consumption: Drinking too much alcohol can increase blood pressure, so it is recommended to limit alcohol intake to no more than one drink per day for women and two drinks per day for men.  Quit smoking: Smoking can raise blood pressure and damage the blood vessels, so quitting smoking can help to lower blood pressure and reduce the risk of heart disease.  Reduce stress: Stress can increase blood pressure, so it is important to find ways to manage stress, such as through relaxation techniques or meditation.  Take medication as prescribed: If lifestyle changes are not enough to lower your blood pressure, your doctor may prescribe medication to help bring it down. It is important to take the medication exactly as prescribed.  It is important to work with your doctor to determine the best treatment plan for your individual needs, and to monitor your blood pressure regularly to ensure that it stays at a healthy level. |
| --- | --- | --- |
| ChatGPT 4.0 | Re 1 | Lifestyle modifications are essential for managing high blood pressure (hypertension) and can sometimes be enough to bring your blood pressure to a normal range. Here are several strategies to help lower your blood pressure:  Lose weight: If you are overweight or obese, losing weight can significantly reduce your blood pressure. Aim for a healthy body mass index (BMI) of 18.5-24.9.  Exercise regularly: Engage in at least 150 minutes of moderate- intensity aerobic exercise or 75 minutes of vigorous-intensity aerobic exercise per week. Activities such as walking, swimming, cycling, or jogging can help lower your blood pressure.  Eat a healthy diet: Consume a diet rich in fruits, vegetables, whole grains, lean proteins, and low-fat dairy products. The DASH (Dietary Approaches to Stop Hypertension) diet is specifically designed to help lower blood pressure.  Reduce sodium intake: Aim for less than 2,300 mg of sodium per day, and ideally, try to consume no more than 1,500 mg daily. Limit processed and restaurant foods, which are often high in sodium, and |

|  |  | use herbs and spices to flavor your meals instead of salt. |
| --- | --- | --- |
|  |  | Limit alcohol consumption: For men, limit alcohol intake to two drinks per day, and for women, no more than one drink per day. Excessive alcohol consumption can raise blood pressure. |
|  |  | Manage stress: Practice stress-reducing techniques such as meditation, yoga, deep breathing exercises, or progressive muscle relaxation. Develop healthy coping mechanisms to manage stress and avoid unhealthy habits like smoking or overeating. |
|  |  | Quit smoking: Smoking contributes to high blood pressure and overall cardiovascular risk. Quitting smoking can improve your blood pressure and overall health. |
|  |  | Maintain a consistent sleep schedule: Aim for 7-8 hours of sleep per night. Inadequate sleep can negatively impact blood pressure. |
|  |  | Monitor your blood pressure regularly: Regularly check your blood pressure at home and keep track of your readings. This can help you identify trends and monitor the effectiveness of lifestyle changes or medications. |
|  |  | Consult a healthcare professional: If lifestyle changes are not sufficient to lower your blood pressure, your doctor may prescribe medication to help manage hypertension. It's essential to follow your healthcare provider's advice and take any prescribed medications as directed. |
|  |  | Please note that it's always important to consult a healthcare professional before making significant changes to your diet, exercise routine, or starting any new treatment. They can provide personalized  guidance based on your specific health needs and circumstances. |
|  |  | Lowering blood pressure is essential for maintaining good cardiovascular health and reducing the risk of heart disease, stroke, and other related conditions. Here are some lifestyle changes and strategies that can help lower blood pressure: |
|  | Re 2 | Eat a healthy diet: Follow the DASH (Dietary Approaches to Stop Hypertension) diet, which emphasizes fruits, vegetables, whole grains, lean proteins, and low-fat dairy products. Reduce your intake of saturated fats, cholesterol, and added sugars. |
|  |  | Reduce sodium intake: Aim to consume no more than 2,300 |

|  |  | milligrams (mg) of sodium per day, or even lower (1,500 mg) if you have high blood pressure. Read food labels, choose low-sodium options, and avoid processed and restaurant foods that tend to be high in sodium.  Exercise regularly: Aim for at least 150 minutes of moderate- intensity aerobic activity or 75 minutes of vigorous-intensity aerobic activity per week. This can include brisk walking, swimming, cycling, or other activities that elevate your heart rate.  Maintain a healthy weight: Losing even a small amount of weight can help lower blood pressure. Talk to your healthcare provider about your ideal weight and work on achieving and maintaining it through diet and exercise.  Limit alcohol consumption: Consume alcohol in moderation, meaning no more than one drink per day for women and two drinks per day for men. Excessive alcohol consumption can raise blood pressure and interfere with blood pressure medications.  Quit smoking: Smoking is a significant risk factor for heart disease, and quitting can help lower blood pressure and improve overall cardiovascular health.  Manage stress: Practice stress management techniques such as deep breathing, meditation, yoga, or progressive muscle relaxation to help reduce blood pressure.  Monitor your blood pressure regularly: Keep track of your blood pressure at home or consult with your healthcare provider to ensure that your blood pressure remains within a healthy range. Regular monitoring can help you identify any trends or changes that might require intervention.  Take prescribed medications as directed: If your healthcare provider has prescribed medication to manage your blood pressure, make sure to take it as directed. Do not skip doses or stop taking the medication without consulting your healthcare provider.  Get enough sleep: Aim for 7-8 hours of quality sleep each night. Poor sleep or sleep disorders, such as sleep apnea, can contribute to high blood pressure.  Limit caffeine intake: Caffeine can cause a temporary spike in blood |
| --- | --- | --- |

|  |  | pressure, especially in individuals who are sensitive to its effects. Monitor your caffeine consumption and consider cutting back if you notice a correlation between your caffeine intake and elevated blood pressure.  Maintain social connections: Engaging in social activities and fostering strong relationships with family and friends can help lower stress levels and contribute to better overall health, including blood pressure management.  Remember that individual results may vary, and it may take time to see significant improvements in blood pressure. It's essential to work closely with your healthcare provider to develop a personalized plan for managing your blood pressure and addressing any underlying  health conditions that may be contributing to high blood pressure. |
| --- | --- | --- |
|  | Re 3 | Lowering your blood pressure can reduce the risk of various health issues, such as heart disease, stroke, and kidney disease. Here are some lifestyle changes that can help lower blood pressure:  Eat a balanced diet: Adopt a heart-healthy diet that includes fruits, vegetables, whole grains, lean proteins, and low-fat dairy products. Limit saturated and trans fats, sodium, and added sugars. The DASH (Dietary Approaches to Stop Hypertension) diet is a well-studied dietary plan designed to help lower blood pressure.  Exercise regularly: Aim for at least 150 minutes of moderate- intensity aerobic activity or 75 minutes of vigorous-intensity aerobic activity per week. Regular physical activity helps lower blood pressure and maintain overall cardiovascular health.  Maintain a healthy weight: Losing excess weight can help lower blood pressure. Even modest weight loss can have a significant impact on reducing blood pressure.  Limit alcohol consumption: If you drink alcohol, do so in moderation. For most adults, that means up to one drink per day for women and up to two drinks per day for men.  Don't smoke: Smoking can damage blood vessels and increase the risk of high blood pressure. If you smoke, quitting is one of the best things you can do for your overall health.  Manage stress: Practice stress reduction techniques such as deep breathing, meditation, yoga, or engaging in hobbies and activities |

|  |  | you enjoy.  Monitor your blood pressure: Keep track of your blood pressure regularly to ensure that it remains within a healthy range. Consult your healthcare provider if your blood pressure remains high or if you have concerns about your readings.  Take prescribed medications: If lifestyle changes alone aren't sufficient to lower your blood pressure, your healthcare provider may prescribe medications to help manage your blood pressure. It's important to take these medications as directed and to continue with the lifestyle changes.  Always consult with your healthcare provider before making any significant changes to your lifestyle or medication regimen, especially if you have a pre-existing medical condition. They can provide personalized advice tailored to your specific needs and  circumstances. |
| --- | --- | --- |

| Question 5 | | I have heart diseases. How to prevent cerebrovascular events? |
| --- | --- | --- |
|  |  | If you have heart disease, there are several steps you can take to prevent cerebrovascular events such as stroke or transient ischemic attack (TIA): |
|  |  | Control your blood pressure: High blood pressure is a major risk factor for cerebrovascular events, so it is important to monitor your blood pressure regularly and take steps to control it. This may include lifestyle changes such as diet and exercise, as well as medications prescribed by your healthcare provider. |
|  |  | Manage your cholesterol levels: High levels of LDL or "bad" cholesterol can increase your risk of cerebrovascular events, so it is important to maintain healthy cholesterol levels through diet, exercise, and medications if needed. |
|  |  | Quit smoking: Smoking is a major risk factor for cerebrovascular events and other heart-related conditions, so quitting smoking is one of the most important steps you can take to protect your health. |
|  | Re 1 |  |
| ChatGPT 3.5 |  | Maintain a healthy weight: Being overweight or obese can increase your risk of cerebrovascular events, so maintaining a healthy weight through diet and exercise is important. |
|  |  | Control your diabetes: Diabetes can increase your risk of cerebrovascular events, so it is important to manage your blood sugar levels through diet, exercise, and medications if needed. |
|  |  | Take prescribed medications: Your healthcare provider may prescribe medications to help prevent cerebrovascular events, such as antiplatelet or anticoagulant medications. |
|  |  | Stay active: Regular physical activity can help reduce your risk of cerebrovascular events, so aim for at least 30 minutes of moderate- intensity exercise most days of the week. |
|  |  | It is important to work with your healthcare provider to develop a plan that is tailored to your individual needs and medical history. |
|  | Re 2 | If you have heart disease, you are at an increased risk for cerebrovascular events such as stroke. Here are some steps you can take to help prevent these events: |
|  |  | Control your blood pressure: High blood pressure is a major risk factor for stroke. Work with your healthcare provider to monitor and |

|  |  | manage your blood pressure through lifestyle changes and medications as needed. |
| --- | --- | --- |
|  |  | Manage diabetes: If you have diabetes, work with your healthcare provider to keep your blood sugar levels under control. High blood sugar levels can increase your risk of stroke. |
|  |  | Maintain a healthy weight: Being overweight or obese can increase your risk of stroke. Losing weight through a healthy diet and regular exercise can help reduce your risk. |
|  |  | Exercise regularly: Regular physical activity can help improve cardiovascular health and reduce your risk of stroke. Aim for at least  150 minutes of moderate-intensity exercise or 75 minutes of vigorous-intensity exercise per week. |
|  |  | Quit smoking: Smoking can increase your risk of stroke and other cardiovascular events. Quitting smoking can help reduce your risk. |
|  |  | Manage stress: Chronic stress can contribute to high blood pressure and increase your risk of stroke. Find ways to manage stress, such as practicing relaxation techniques, getting enough sleep, and engaging in enjoyable activities. |
|  |  | Take medications as prescribed: Your healthcare provider may prescribe medications such as antiplatelet drugs or anticoagulants to help prevent stroke. It is important to take these medications as prescribed. |
|  |  | In addition to these steps, it is important to work closely with your healthcare provider to manage your heart disease and reduce your risk of cerebrovascular events. Regular check-ups, monitoring of your condition, and adjustments to your treatment plan as needed can  all help keep you healthy and reduce your risk of stroke. |
|  |  | People with heart disease are at an increased risk of cerebrovascular events, such as stroke. However, there are steps that can be taken to help prevent these events. Here are some strategies that may help: |
|  | Re 3 | Control blood pressure: High blood pressure is a major risk factor for stroke, so it is important to keep blood pressure under control. This can be achieved through lifestyle changes, such as a healthy diet, regular exercise, and limiting alcohol intake, as well as medication prescribed by a doctor. |

|  |  | Manage cholesterol levels: High cholesterol can increase the risk of stroke, so it is important to manage cholesterol levels through a healthy diet and medication prescribed by a doctor.  Control diabetes: Diabetes can increase the risk of stroke, so it is important to keep blood sugar levels under control through diet, exercise, and medication prescribed by a doctor.  Quit smoking: Smoking is a major risk factor for stroke, so quitting smoking is essential for reducing the risk of cerebrovascular events.  Maintain a healthy weight: Being overweight or obese can increase the risk of stroke, so maintaining a healthy weight through a healthy diet and regular exercise is important.  Exercise regularly: Regular exercise can help to improve heart health and reduce the risk of stroke.  Take medication as prescribed: If prescribed medication for heart disease or stroke prevention, it is important to take it exactly as prescribed by a doctor.  It is also important to have regular check-ups with a doctor to monitor and manage any underlying health conditions and to identify and address any new risk factors for cerebrovascular events. |
| --- | --- | --- |
| ChatGPT 4.0 | Re 1 | If you have heart disease, you are at an increased risk of cerebrovascular events, such as strokes or transient ischemic attacks (TIAs). To minimize this risk, it's essential to manage your heart condition and adopt a heart-healthy lifestyle. Here are some strategies to help prevent cerebrovascular events:  Manage your heart condition: Follow your healthcare provider's recommendations for managing your specific heart disease, which may include medications, medical procedures, or lifestyle modifications.  Control high blood pressure: Consistently high blood pressure is a significant risk factor for cerebrovascular events. Maintain a healthy lifestyle, and follow your doctor's recommendations for managing your blood pressure, which may include medication.  Maintain a healthy weight: Excess weight can increase your risk of high blood pressure and other cardiovascular issues. Aim for a healthy body mass index (BMI) of 18.5-24.9. |

|  |  | Eat a heart-healthy diet: Consume a diet rich in fruits, vegetables, whole grains, lean proteins, and low-fat dairy products. The DASH (Dietary Approaches to Stop Hypertension) diet and the Mediterranean diet are both known for their heart-healthy benefits.  Exercise regularly: Engage in at least 150 minutes of moderate- intensity aerobic exercise or 75 minutes of vigorous-intensity aerobic exercise per week, as recommended by the American Heart Association. Consult your healthcare provider before starting a new exercise program, especially if you have heart disease.  Quit smoking: Smoking increases your risk of both heart disease and cerebrovascular events. Quitting smoking can significantly reduce these risks.  Limit alcohol consumption: Excessive alcohol consumption can raise blood pressure and increase the risk of cerebrovascular events. Limit alcohol intake to one drink per day for women and up to two drinks per day for men.  Control diabetes: If you have diabetes, maintain proper blood sugar levels through diet, exercise, and medications, as prescribed by your healthcare provider.  Monitor and manage cholesterol levels: High cholesterol can contribute to atherosclerosis, which can lead to cerebrovascular events. Follow your healthcare provider's recommendations for managing cholesterol levels, which may include medications, dietary changes, and exercise.  Consider antiplatelet or anticoagulant medications: If you are at high risk for cerebrovascular events, your healthcare provider may recommend medications such as aspirin or other antiplatelet or anticoagulant drugs to help prevent blood clots.  It's essential to work closely with your healthcare provider to develop a personalized plan for managing your heart disease and reducing the risk of cerebrovascular events. Regular check-ups and monitoring can help ensure your plan remains effective and up-to-date with your changing health needs. |
| --- | --- | --- |
|  | Re 2 | I'm not a doctor, but I can provide you with some general information  on how to potentially reduce the risk of cerebrovascular events, such as strokes or transient ischemic attacks (TIAs), if you have heart |

|  |  | disease. It's essential to consult with a healthcare professional for personalized advice tailored to your specific situation.  Control blood pressure: High blood pressure is a significant risk factor for stroke. Monitor your blood pressure regularly and follow your healthcare provider's recommendations to keep it in a healthy range.  Manage cholesterol: High cholesterol levels can contribute to the narrowing of blood vessels, increasing your risk of stroke. Eat a healthy diet, exercise regularly, and take medications as prescribed to help manage cholesterol levels.  Maintain a healthy weight: Being overweight or obese can increase your risk of heart disease and stroke. Aim to maintain a healthy weight through a balanced diet and regular exercise.  Exercise regularly: Physical activity can help lower blood pressure, improve circulation, and strengthen your heart. Aim for at least 150 minutes of moderate-intensity aerobic activity or 75 minutes of vigorous-intensity aerobic activity per week.  Eat a heart-healthy diet: Consume a diet rich in fruits, vegetables, whole grains, lean proteins, and low-fat dairy products. Limit your intake of saturated fats, trans fats, cholesterol, salt, and added sugars.  Don't smoke: Smoking increases the risk of stroke and heart disease. If you smoke, quitting is one of the best things you can do for your health.  Limit alcohol consumption: Excessive alcohol intake can raise blood pressure and contribute to stroke risk. If you choose to drink, do so in moderation—up to one drink per day for women and up to two drinks per day for men.  Manage diabetes: High blood sugar can damage blood vessels, increasing the risk of stroke. If you have diabetes, work with your healthcare provider to manage your blood sugar levels effectively.  Take medications as prescribed: If you're on medications for heart disease, blood pressure, cholesterol, or other conditions, take them as prescribed and follow your healthcare provider's guidance.  Monitor and manage atrial fibrillation (AFib): AFib is an irregular |
| --- | --- | --- |

|  |  | heartbeat that can increase the risk of stroke. If you have AFib, work with your healthcare provider to manage the condition and reduce your risk of stroke. |
| --- | --- | --- |
|  |  | Remember, it's crucial to consult with your healthcare provider about your specific situation and follow their recommendations for managing your heart disease and reducing your risk of cerebrovascular events. |
|  |  | I am not a doctor, but I can provide you with some general advice on how to reduce the risk of cerebrovascular events, such as strokes, especially if you have heart disease. It is important to consult with your healthcare provider to develop a personalized plan based on your specific needs and medical history. Here are some general strategies that may help: |
|  |  | Manage blood pressure: High blood pressure is a significant risk factor for both heart disease and stroke. Maintain a healthy lifestyle, including a balanced diet, regular exercise, stress management, and adhering to any prescribed blood pressure medications to help control your blood pressure. |
|  |  | Control cholesterol: High cholesterol levels can contribute to plaque buildup in your arteries, increasing your risk of stroke. A heart- healthy diet, regular exercise, and prescribed medications can help keep your cholesterol levels in check. |
|  | Re 3 | Maintain a healthy weight: Excess weight can contribute to high blood pressure, high cholesterol, and increased risk of heart disease and stroke. Aim to achieve and maintain a healthy weight through diet and exercise. |
|  |  | Manage diabetes: If you have diabetes, it is crucial to manage your blood sugar levels, as poorly controlled diabetes can increase your risk of heart disease and stroke. |
|  |  | Don't smoke: Smoking increases your risk of heart disease, stroke, and other health issues. If you smoke, quitting is one of the best things you can do for your health. |
|  |  | Limit alcohol consumption: If you drink alcohol, do so in moderation. Excessive alcohol consumption can increase your risk of stroke and other health problems. |
|  |  | Exercise regularly: Engage in regular physical activity to help |

|  |  | maintain cardiovascular health, manage weight, and reduce your risk of stroke.  Take prescribed medications: If you have been prescribed medications for heart disease, blood pressure, cholesterol, or other conditions, it is essential to take them as directed by your healthcare provider.  Monitor for atrial fibrillation: Atrial fibrillation, an irregular and often rapid heart rate, can increase the risk of stroke. If you have atrial fibrillation, work with your healthcare provider to manage this condition, which may include medications or medical procedures.  It's essential to work closely with your healthcare provider to develop a personalized plan for managing your heart disease and reducing your risk of cerebrovascular events. Regular check-ups and communication with your healthcare team can help ensure you're on  the right track. |
| --- | --- | --- |

| Question 6 | | How many daily steps do I need to prevent stroke? |
| --- | --- | --- |
| ChatGPT 3.5 | Re 1 | The number of daily steps needed to prevent stroke may vary depending on several factors such as age, sex, health status, and physical fitness level. However, studies have shown that walking or engaging in other moderate-intensity physical activity for at least 30 minutes a day, most days of the week, can help reduce the risk of stroke.  It is recommended to aim for a daily goal of 10,000 steps or more, which is roughly equivalent to 30 minutes of moderate-intensity physical activity per day. However, any amount of physical activity is better than none, so starting with smaller goals and gradually increasing your activity level can be a good way to establish a regular exercise routine.  In addition to walking or other forms of aerobic exercise, it is also important to engage in strength-training exercises that target major muscle groups, such as the legs, arms, back, and abdomen. This can help improve overall fitness, reduce the risk of falls, and improve overall health and well-being.  It is important to speak with your healthcare provider before starting or changing your exercise routine, especially if you have a history of stroke or other medical conditions. |
|  | Re 2 | There is no specific number of daily steps that can guarantee the prevention of stroke. However, regular physical activity, including walking, has been shown to reduce the risk of stroke.  According to the American Heart Association, adults should aim for at least 150 minutes of moderate-intensity aerobic exercise or 75 minutes of vigorous-intensity aerobic exercise per week. This can include activities such as brisk walking, cycling, swimming, or other forms of aerobic exercise.  In terms of steps, a goal of 10,000 steps per day is often recommended as a general guideline for overall health and fitness. However, the number of steps needed to achieve the recommended amount of physical activity can vary depending on factors such as age, fitness level, and health status.  The most important thing is to find a physical activity routine that you enjoy and can stick to regularly. Talk to your healthcare provider about an exercise plan that is appropriate for your individual needs  and health status. |

|  | Re 3 | There is no specific number of daily steps that can guarantee stroke prevention as it can depend on various factors such as age, sex, medical history, and overall health. However, generally, it is recommended to aim for at least 150 minutes of moderate-intensity aerobic exercise or 75 minutes of vigorous-intensity aerobic exercise per week. Walking is a great form of moderate-intensity aerobic exercise.  In terms of steps, a good goal to aim for is 10,000 steps per day, which is roughly equivalent to walking 5 miles. However, this number can vary based on individual factors and goals.  It's important to note that regular physical activity, including walking, has been shown to help reduce the risk of stroke by improving cardiovascular health, reducing blood pressure, and lowering the risk of diabetes and other chronic conditions. Therefore, incorporating regular physical activity, including walking, into your  daily routine can be a great way to help prevent stroke. |
| --- | --- | --- |
| ChatGPT 4.0 | Re 1 | There is no specific number of daily steps that can guarantee the prevention of a stroke. However, engaging in regular physical activity can significantly reduce the risk of stroke and other cardiovascular diseases. The American Heart Association (AHA) recommends at least 150 minutes of moderate-intensity aerobic exercise or 75 minutes of vigorous-intensity aerobic exercise per week, or a combination of both, for adults. This translates to about 30 minutes of physical activity on most days of the week.  Walking is a great form of moderate-intensity aerobic exercise that can help you achieve these recommendations. Aiming for around 10,000 steps per day is a common goal for promoting general health and well-being. However, the optimal number of daily steps can vary depending on factors such as age, fitness level, and overall health.  It's important to remember that any increase in physical activity can be beneficial, even if you don't reach the recommended 10,000 steps per day. For individuals who are currently sedentary, starting with a smaller goal, such as 4,000 to 6,000 steps per day, and gradually increasing the number of steps over time can still provide significant health benefits.  Please consult your healthcare provider before starting a new exercise routine, especially if you have any pre-existing health conditions or concerns. They can help you determine the most  appropriate level of physical activity based on your personal health |

|  |  | needs and circumstances. |
| --- | --- | --- |
|  | Re 2 | There isn't a specific number of daily steps proven to prevent stroke for everyone, as individual needs may vary. However, engaging in regular physical activity is associated with a reduced risk of stroke and other cardiovascular diseases. The American Heart Association recommends that adults aim for at least 150 minutes of moderate- intensity aerobic activity or 75 minutes of vigorous-intensity aerobic activity per week, which can be broken down into smaller sessions.  Walking can be a great way to achieve these recommendations. Aiming for 10,000 steps per day is a popular goal that many people use to increase their physical activity. However, it is important to note that any increase in physical activity can be beneficial, especially for those who are currently sedentary or have limited mobility. If you are unable to reach 10,000 steps, don't be discouraged; start with a smaller goal and gradually increase your steps over time.  Before starting any new exercise program, it's important to consult with your healthcare provider, especially if you have heart disease or other health conditions. They can help you determine the most appropriate and safe level of physical activity for your specific  situation. |
|  | Re 3 | There isn't a specific number of daily steps that can guarantee the prevention of a stroke, as stroke risk depends on various factors, such as genetics, age, overall health, and lifestyle choices. However, engaging in regular physical activity is an essential component of a healthy lifestyle and can help reduce the risk of stroke and other cardiovascular diseases.  The American Heart Association recommends that adults engage in at least 150 minutes of moderate-intensity aerobic activity or 75 minutes of vigorous-intensity aerobic activity per week, spread across multiple days. This can be achieved through various forms of exercise, including walking, which is a low-impact and accessible form of physical activity for many people.  Some studies suggest that taking around 10,000 steps per day may help improve overall health and reduce the risk of chronic diseases, although the optimal number of steps can vary based on individual factors such as age, fitness level, and personal health goals.  To reduce your risk of stroke and promote overall cardiovascular health, consider the following: |

|  |  | Engage in regular physical activity, aiming for at least 150 minutes of moderate-intensity or 75 minutes of vigorous-intensity aerobic activity per week.  Incorporate a variety of exercises into your routine, including aerobic activities, strength training, and flexibility exercises.  Gradually increase your daily step count, aiming for a goal that is challenging but achievable based on your current fitness level and health status.  Use a pedometer, smartphone app, or fitness tracker to monitor your daily steps and motivate yourself to stay active.  Keep in mind that individual needs and goals may vary, and it's essential to consult with your healthcare provider before starting a new exercise program, especially if you have pre-existing health conditions or concerns. They can help you develop a personalized  plan that is appropriate for your specific situation and health goals. |
| --- | --- | --- |

| Question 7 | | What kind of exercise can I do after stroke? |
| --- | --- | --- |
| ChatGPT 3.5 | Re 1 | After a stroke, it is important to work closely with a healthcare provider or physical therapist to develop an exercise program that is tailored to your individual needs and abilities. Depending on the severity of the stroke and the extent of any physical impairments, exercises may focus on improving strength, coordination, balance, and mobility.  Some examples of exercises that may be recommended after a stroke include:  Range of motion exercises: These exercises involve moving your arms, legs, and other affected body parts through a full range of motion to help improve flexibility and prevent stiffness.  Strength training exercises: These exercises involve using weights, resistance bands, or your own body weight to help build strength in the affected muscles.  Balance exercises: These exercises may involve standing on one leg, using a balance board, or practicing other activities that challenge your balance and coordination.  Aerobic exercise: Depending on your physical abilities, you may be able to engage in activities such as walking, cycling, or swimming to help improve cardiovascular health and overall fitness.  Functional training: These exercises involve practicing everyday activities such as dressing, cooking, or grooming to help improve independence and quality of life.  It is important to work with a healthcare provider or physical therapist to develop an exercise program that is safe and appropriate for your individual needs and abilities. Exercise should be done in a gradual and progressive manner, and any signs of fatigue or  discomfort should be reported to your healthcare provider. |
|  | Re 2 | After a stroke, it is important to work with your healthcare provider and a rehabilitation team to develop an exercise plan that is safe and appropriate for your individual needs and abilities. Here are some examples of exercises that may be recommended:  Range of motion exercises: These exercises involve moving the affected limbs and joints through their full range of motion to help maintain flexibility and prevent muscle stiffness. |

|  |  | Aerobic exercises: These exercises involve sustained physical activity that increases your heart rate and breathing rate. Examples may include walking, cycling, swimming, or using a stationary bike.  Strength training exercises: These exercises involve using resistance to build strength in the muscles. Examples may include using weights or resistance bands.  Balance and coordination exercises: These exercises involve challenging your balance and coordination to help improve your ability to perform daily activities and reduce the risk of falls.  Functional exercises: These exercises involve practicing everyday activities such as getting up from a chair or bed, reaching for objects, and walking on different surfaces.  It is important to start with low-intensity exercises and gradually increase intensity and duration as your fitness level improves. Your healthcare provider and rehabilitation team can provide guidance on  how to safely and effectively exercise after a stroke. |
| --- | --- | --- |
|  | Re 3 | After a stroke, it's important to consult with a healthcare professional or physical therapist to determine what exercises are safe and appropriate for your specific needs and abilities. However, here are some general types of exercise that may be recommended:  Range of motion exercises: These exercises help to maintain or improve flexibility and mobility of joints and muscles. Examples include gentle stretches, ankle and wrist rotations, and shoulder shrugs.  Strengthening exercises: These exercises help to build strength in muscles that may have been weakened by stroke. Examples include resistance band exercises, weight lifting, and isometric exercises.  Aerobic exercises: These exercises help to improve cardiovascular health and overall fitness. Examples include walking, cycling, swimming, and low-impact aerobics.  Balance exercises: These exercises help to improve balance and reduce the risk of falls. Examples include standing on one leg, heel- to-toe walking, and Tai Chi.  Functional exercises: These exercises focus on performing activities |

|  |  | of daily living, such as getting dressed, cooking, and cleaning. A physical therapist may help you practice these activities in a safe and controlled environment.  It's important to start with exercises that are appropriate for your current level of ability and gradually increase the intensity and duration of exercise over time. It's also important to listen to your body and avoid any exercises that cause pain or discomfort. |
| --- | --- | --- |
|  |  | After a stroke, it's crucial to consult with your healthcare provider, physical therapist, or occupational therapist to develop an individualized exercise program tailored to your specific needs, abilities, and recovery goals. The types of exercises you can do after a stroke will depend on the severity of the stroke, your overall health, and the degree of your functional impairments. Some common types of exercises that may be recommended during stroke rehabilitation include: |
|  |  | Range of motion exercises: These exercises help maintain or improve flexibility, reduce stiffness, and prevent joint contractures. They involve moving the affected limbs through their full range of motion, either passively (with assistance) or actively (on your own). |
| ChatGPT 4.0 | Re 1 | Strengthening exercises: After a stroke, muscle weakness is common, especially on one side of the body. Strengthening exercises, such as resistance training with elastic bands or light weights, can help rebuild muscle strength and coordination. |
|  |  | Balance and coordination exercises: Improving balance and coordination is essential for preventing falls and regaining functional independence. Exercises like standing on one leg, tandem walking (placing one foot directly in front of the other), or practicing sit-to- stand transitions can help improve balance and coordination. |
|  |  | Aerobic exercises: As your recovery progresses, incorporating low- impact aerobic exercises like walking, cycling, swimming, or using a stationary bike or elliptical trainer can help improve cardiovascular fitness and overall endurance. |
|  |  | Gait training: If you have difficulty walking after a stroke, gait training exercises with a physical therapist can help improve your walking pattern, speed, and stability. This may involve the use of assistive devices, such as canes, walkers, or ankle-foot orthoses. |
|  |  | Functional task-specific training: Practicing daily activities, like |

|  |  | dressing, grooming, or using utensils, can help improve fine motor skills and support your return to normal daily routines. |
| --- | --- | --- |
|  |  | Group exercise classes: Participating in group exercise classes designed for stroke survivors, such as adapted yoga, tai chi, or aquatic therapy, can provide physical, social, and emotional benefits. |
|  |  | Always consult your healthcare team before starting any new exercise program, and follow their guidance regarding the types, intensity, and duration of exercises that are most appropriate for your specific situation. Remember that recovery after a stroke can take time, and progress may be gradual. It's essential to be patient, persistent, and maintain open communication with your healthcare  providers throughout the rehabilitation process. |
|  |  | I'm not a healthcare professional, but I can offer some general information on exercises that might be helpful after a stroke. It's crucial to consult with your healthcare provider, a physical therapist, or an occupational therapist before starting any exercise program, as they can assess your individual needs and recommend the most appropriate and safe exercises for you. |
|  |  | After a stroke, exercises often focus on improving strength, flexibility, balance, and coordination. Here are some types of exercises that might be suggested, depending on your specific situation and recovery goals: |
|  | Re 2 | Range of motion exercises: These exercises help improve joint flexibility and reduce stiffness. They may involve moving your limbs through their full range of motion, either with assistance or on your own. |
|  |  | Strengthening exercises: After a stroke, some muscles may be weaker than others. Strengthening exercises can help rebuild muscle strength and improve functional abilities. These might include using resistance bands, bodyweight exercises, or light weights. |
|  |  | Balance and coordination exercises: Improving balance and coordination can help reduce the risk of falls and improve mobility. Exercises might involve standing on one foot, practicing shifting weight from one leg to another, or using balance boards. |
|  |  | Aerobic exercises: As you recover and gain strength, your healthcare provider may recommend incorporating aerobic exercises, such as walking, cycling, or swimming, to improve cardiovascular fitness |

|  |  | and overall health. |
| --- | --- | --- |
|  |  | Stretching exercises: Stretching can help maintain flexibility, reduce muscle stiffness, and improve posture. It's essential to stretch gently and follow the guidance of your healthcare provider or therapist. |
|  |  | Task-specific training: Practicing specific tasks or activities, such as grasping objects or climbing stairs, can help improve your ability to perform daily activities. |
|  |  | Remember to always consult with your healthcare provider, physical therapist, or occupational therapist before starting any exercise program after a stroke. They will be able to create a personalized plan tailored to your specific needs and recovery goals. Also, be patient with your progress, as recovery can take time and may require  consistent effort. |
|  |  | I'm not a doctor, but I can offer you some general information on exercises that may be suitable for stroke survivors. It's crucial to consult with your healthcare provider, physical therapist, or a qualified professional before starting any exercise program, as they can tailor recommendations based on your specific needs, abilities, and rehabilitation goals. |
|  |  | Post-stroke exercises typically focus on improving strength, flexibility, balance, and coordination, as well as addressing any specific impairments you may have. Some examples of exercises that might be included in a post-stroke rehabilitation program are: |
|  | Re 3 | Range-of-motion exercises: These exercises help maintain or improve joint flexibility and can involve gentle stretching and movements of the affected limbs. |
|  |  | Strengthening exercises: Resistance training, using body weight or resistance bands, can help build strength in weakened muscles. |
|  |  | Balance and coordination exercises: Activities such as standing on one leg, weight shifting, or practicing various walking patterns can help improve balance and coordination. |
|  |  | Aerobic exercises: Low-impact aerobic activities like walking, cycling, or swimming can help improve cardiovascular fitness and overall endurance. Start slow and gradually increase intensity and duration as your abilities and fitness level improve. |

|  |  | Functional training: Practicing daily activities like getting in and out of a chair, climbing stairs, or picking up objects can help improve functional independence.  Speech and cognitive exercises: If you have speech or cognitive impairments, working with a speech therapist on specific exercises can help improve communication and cognitive skills.  It's essential to work closely with your rehabilitation team, which may include physical therapists, occupational therapists, and speech therapists, to develop a personalized plan that addresses your specific needs and goals. Remember to communicate any concerns or difficulties you may be experiencing so that your healthcare team can make adjustments to your exercise program as needed. |
| --- | --- | --- |

| Question 8 | | Would hypertension lead to brain bleed? |
| --- | --- | --- |
| ChatGPT 3.5 | Re 1 | Yes, hypertension (high blood pressure) is a major risk factor for brain bleeding or hemorrhagic stroke. This occurs when a weakened blood vessel in the brain ruptures and bleeds into the surrounding brain tissue. Hypertension puts increased pressure on blood vessels, making them more prone to rupture and increasing the risk of brain bleeding.  If left uncontrolled, hypertension can cause damage to blood vessels in the brain over time, leading to a higher risk of brain bleeding. It is important to manage hypertension through lifestyle changes and medications prescribed by a healthcare provider to reduce the risk of complications such as brain bleeding. Monitoring blood pressure regularly and seeking medical attention if any concerning symptoms  arise is also important in preventing and managing brain bleeding. |
|  | Re 2 | Yes, hypertension (high blood pressure) can increase the risk of brain bleed, also known as intracerebral hemorrhage. Chronic hypertension can weaken the small blood vessels in the brain, causing them to rupture and leak blood into the surrounding brain tissue. The bleeding can cause damage to the brain tissue and can be life-threatening.  The risk of intracerebral hemorrhage increases as blood pressure levels rise. Therefore, it is important to control hypertension to reduce the risk of brain bleed. This can be achieved through lifestyle changes such as maintaining a healthy weight, eating a balanced diet, reducing sodium intake, limiting alcohol consumption, and engaging in regular physical activity. Your healthcare provider may also prescribe medications to help lower your blood pressure.  If you have hypertension, it is important to monitor your blood pressure regularly and work closely with your healthcare provider to manage your condition and reduce your risk of complications such as brain bleed. |
|  | Re 3 | Yes, hypertension (high blood pressure) can increase the risk of brain bleed, also known as a hemorrhagic stroke. High blood pressure can cause damage to the small blood vessels in the brain, making them more prone to rupturing and causing bleeding. Over time, uncontrolled high blood pressure can weaken the blood vessels and increase the risk of a hemorrhagic stroke.  Hemorrhagic strokes account for approximately 15% of all strokes, but they tend to be more severe and have a higher mortality rate than ischemic strokes (caused by a blockage in a blood vessel in the |

|  |  | brain).  Managing high blood pressure is an important way to help reduce the risk of a hemorrhagic stroke. This can be done through lifestyle changes such as eating a healthy diet, exercising regularly, maintaining a healthy weight, quitting smoking, and limiting alcohol  intake, as well as medication prescribed by a healthcare professional. |
| --- | --- | --- |
| ChatGPT 4.0 | Re 1 | Hypertension, or high blood pressure, can increase the risk of a brain bleed, also known as a hemorrhagic stroke. A hemorrhagic stroke occurs when a blood vessel in the brain ruptures, causing bleeding into the surrounding brain tissue. There are two main types of hemorrhagic strokes: intracerebral hemorrhage and subarachnoid hemorrhage.  Intracerebral hemorrhage is the most common type of hemorrhagic stroke, where bleeding occurs within the brain tissue. Hypertension is a major risk factor for this type of stroke, as consistently high blood pressure can weaken and damage the walls of blood vessels, making them more susceptible to rupture.  Subarachnoid hemorrhage is less common and occurs when bleeding happens in the subarachnoid space, which is the area between the brain and the thin tissues that cover it. While hypertension can also be a risk factor for subarachnoid hemorrhage, other factors such as aneurysms, arteriovenous malformations, or head injuries can contribute to the risk as well.  Maintaining healthy blood pressure levels is essential for reducing the risk of both ischemic and hemorrhagic strokes. Lifestyle modifications, such as eating a balanced diet, exercising regularly, managing stress, maintaining a healthy weight, limiting alcohol consumption, and quitting smoking, can help control blood pressure. In some cases, medication may also be necessary to manage hypertension effectively. It is crucial to work closely with your healthcare provider to monitor and manage your blood pressure and  reduce the risk of stroke and other complications. |
|  | Re 2 | Hypertension, or high blood pressure, can potentially lead to a brain bleed, also known as a hemorrhagic stroke. There are two main types of hemorrhagic strokes: intracerebral hemorrhage and subarachnoid hemorrhage.  Intracerebral hemorrhage: This occurs when a blood vessel within the brain tissue itself ruptures, causing bleeding into the surrounding brain tissue. Hypertension is a major risk factor for intracerebral |

|  |  | hemorrhage, as it can weaken and damage the walls of blood vessels over time.  Subarachnoid hemorrhage: This type of brain bleed occurs when there is bleeding into the space between the brain and the surrounding membrane (the subarachnoid space). This is often due to the rupture of an aneurysm, a weak or bulging spot on a blood vessel's wall. While hypertension can contribute to the development of aneurysms, there are other factors that can also increase the risk, such as smoking, excessive alcohol consumption, and family history.  It is essential to manage hypertension to reduce the risk of brain bleeds and other health complications. To do so, you should:  Monitor your blood pressure regularly and follow your healthcare provider's recommendations for maintaining a healthy blood pressure level.  Adopt a heart-healthy diet that includes fruits, vegetables, whole grains, lean proteins, and low-fat dairy, while limiting saturated and trans fats, sodium, and added sugars.  Exercise regularly, aiming for at least 150 minutes of moderate- intensity aerobic activity or 75 minutes of vigorous-intensity aerobic activity per week.  Maintain a healthy weight through a balanced diet and regular physical activity.  Don't smoke, and if you do smoke, quit.  Limit alcohol consumption to moderate levels (up to one drink per day for women and up to two drinks per day for men).  Take medications as prescribed by your healthcare provider to manage blood pressure and other related conditions.  It's crucial to consult with your healthcare provider about your specific situation and follow their recommendations for managing hypertension and reducing the risk of brain bleeds and other  complications. |
| --- | --- | --- |
|  | Re 3 | Hypertension, or high blood pressure, can be a risk factor for a brain bleed, also known as a hemorrhagic stroke. A hemorrhagic stroke occurs when a blood vessel in the brain ruptures and bleeds, causing damage to the surrounding brain tissue.  Chronic hypertension can weaken blood vessel walls, making them more susceptible to rupture. Additionally, high blood pressure can contribute to the formation of aneurysms, which are weak, bulging areas in blood vessels that can burst and result in a brain bleed. |

|  |  | It's important to note that while hypertension is a significant risk factor, not everyone with high blood pressure will experience a brain bleed. Other risk factors for hemorrhagic stroke include:  Age: Older individuals are at higher risk.  Family history: A family history of hemorrhagic stroke or aneurysms can increase your risk.  Smoking: Smoking damages blood vessels and increases the risk of stroke.  Excessive alcohol consumption: Heavy drinking can contribute to high blood pressure and increase the risk of hemorrhagic stroke.  Use of blood-thinning medications: Certain medications, such as anticoagulants, can increase the risk of bleeding in the brain.  Medical conditions: Conditions like arteriovenous malformations (AVMs) or amyloid angiopathy can also increase the risk of brain bleeds.  Managing your blood pressure and addressing other modifiable risk factors can help reduce your risk of a brain bleed. This may include adopting a healthy lifestyle, such as eating a balanced diet, exercising regularly, maintaining a healthy weight, quitting smoking, and limiting alcohol intake. In some cases, medications may be prescribed to help manage high blood pressure.  It's essential to consult with your healthcare provider to develop a personalized plan for managing your blood pressure and reducing your risk of hemorrhagic stroke. Regular check-ups and monitoring  can help ensure that you're on the right track. |
| --- | --- | --- |

| Question 9 | | I used to take aspirin, but I had a neurosurgical operation recently.  When can I retreat aspirin? |
| --- | --- | --- |
| ChatGPT 3.5 | Re 1 | Aspirin is a blood-thinning medication that can increase the risk of bleeding, especially after a neurosurgical operation. Therefore, the decision to restart aspirin after surgery should be made by your healthcare provider based on several factors, including the type of surgery, the extent of bleeding during the surgery, and your individual medical history and risk factors.  In general, your healthcare provider may recommend delaying the restart of aspirin for a period of time after surgery to allow for proper healing and to minimize the risk of bleeding complications. The exact duration of this delay may vary depending on the individual case and should be determined by your healthcare provider.  It is important to follow your healthcare provider's instructions regarding the use of aspirin and any other medications after surgery to minimize the risk of complications and promote proper healing. If you have any concerns or questions about when to restart aspirin, it  is important to discuss them with your healthcare provider. |
|  | Re 2 | It is important to follow the recommendations of your healthcare provider regarding when to restart aspirin after a neurosurgical operation, as the timing may vary depending on your individual circumstances.  In general, aspirin is often discontinued before surgery to reduce the risk of bleeding complications. The decision to restart aspirin after surgery will depend on factors such as the type of surgery, the extent of the surgery, and the presence of other medical conditions.  Your healthcare provider will monitor your recovery and may recommend restarting aspirin when it is safe to do so. This may be several days or weeks after the surgery, depending on the specific circumstances. They may also recommend a different dose or schedule for taking aspirin after surgery.  It is important to discuss any concerns or questions about restarting aspirin with your healthcare provider. They can provide personalized guidance based on your individual medical history and current condition. |
|  | Re 3 | The decision to restart aspirin after a neurosurgical operation should be made by a healthcare professional based on several factors, including the reason why you were taking aspirin in the first place,  the type of surgery you had, and your risk of bleeding. |

|  |  | Aspirin is often prescribed to help prevent blood clots and reduce the risk of stroke and heart attack. However, it can also increase the risk of bleeding, which may be a concern after neurosurgical operations.  In general, it's recommended to avoid aspirin and other blood- thinning medications for a certain period before and after surgery to reduce the risk of bleeding. The exact timing may depend on the type of surgery you had and your individual risk factors.  Your healthcare professional will advise you on when it's safe to restart aspirin after your neurosurgical operation. They may also consider alternative medications or strategies to manage your risk of blood clots and stroke. It's important to follow their recommendations and not to start taking aspirin again without their  guidance. |
| --- | --- | --- |
| ChatGPT 4.0 | Re 1 | I am not a doctor, but I can provide you with general information about resuming aspirin after neurosurgical procedures. The decision to restart aspirin after surgery depends on several factors, including the type of surgery, your individual risk factors for bleeding and clotting, and the recommendations of your healthcare team.  In general, after neurosurgical procedures, there is often a concern for the risk of bleeding. Therefore, your surgeon and healthcare team will carefully evaluate the benefits and risks of resuming aspirin. They will typically provide you with guidance on when and if you can restart aspirin based on your specific circumstances.  It is essential to follow your healthcare provider's recommendations and not restart aspirin without their approval. Make sure to discuss your medical history, including your history of taking aspirin and the reasons for taking it, with your surgeon and healthcare team. They will provide you with personalized guidance on managing your medications during the postoperative period.  If you have concerns or questions about resuming aspirin after your neurosurgical operation, please consult your healthcare provider for the most accurate and personalized advice. |
|  | Re 2 | I am not a doctor, but I can provide some general information on this topic. Resuming aspirin or other blood-thinning medications after neurosurgery depends on various factors, including the type of surgery, your overall health, and the recommendations of your healthcare team. |

|  |  | It is essential to discuss this issue with your neurosurgeon, primary care physician, or another healthcare professional involved in your care. They will be able to assess your individual situation and provide specific guidance on when it may be safe to restart aspirin or other medications. |
| --- | --- | --- |
|  |  | Do not make any changes to your medication regimen without consulting your healthcare provider, as doing so may pose risks to your health and recovery. |
|  |  | I'm not a doctor, but I can offer some general advice on this topic. It's crucial that you consult with your healthcare provider or neurosurgeon before resuming aspirin or any other medication after a neurosurgical operation. They will be able to provide you with specific recommendations based on your medical history, the type of surgery you had, and your current health status. |
|  | Re 3 | Aspirin has blood-thinning effects, which can increase the risk of bleeding. Your doctor will need to assess your individual risk factors and the potential benefits of resuming aspirin therapy, taking into consideration the healing process and any potential complications related to your surgery. |
|  |  | In some cases, your doctor may recommend that you wait for a specific period before resuming aspirin or may suggest an alternative medication based on your individual needs. It's essential to follow your healthcare provider's recommendations and not to make any  changes to your medication regimen without their guidance. |

| Question 10 | | Will I pass my intracranial aneurysm to my children? |
| --- | --- | --- |
|  |  | Intracranial aneurysms are not directly inherited in a simple manner, meaning that they do not follow a predictable pattern of inheritance like some genetic disorders. However, there are some factors that may increase the likelihood of aneurysm formation, such as family history and certain genetic conditions. |
|  |  | If you have a family history of intracranial aneurysms, your children may have a slightly increased risk of developing one themselves. However, this risk is still relatively small and varies depending on  the specifics of your family history. |
|  | Re 1 |  |
|  |  | It is important to note that most people with a family history of intracranial aneurysms do not develop an aneurysm themselves. In addition, many cases of intracranial aneurysms occur in individuals with no family history of the condition. |
| ChatGPT 3.5 |  | If you are concerned about your children's risk of developing an intracranial aneurysm, it may be helpful to speak with a genetic counselor or other healthcare professional who can provide personalized information and guidance based on your specific family  history and medical background. |
|  |  | Intracranial aneurysms are not typically inherited in a simple, predictable way. However, there may be a genetic predisposition to developing aneurysms. Studies have shown that having a first-degree relative (such as a parent or sibling) with an intracranial aneurysm may increase the risk of developing an aneurysm yourself. |
|  | Re 2 | It is important to note that the overall risk of developing an intracranial aneurysm is relatively low, even with a family history. Other factors such as age, smoking, hypertension, and certain medical conditions can also increase the risk of developing an aneurysm. |
|  |  | If you have a history of intracranial aneurysm in your family, it is important to talk to your healthcare provider about your individual risk and any recommended screening or preventive measures. Your healthcare provider may recommend screening for aneurysms if you have a family history, especially if you have other risk factors or  symptoms. |
|  | Re 3 | Intracranial aneurysms are not typically inherited in a simple manner. Although there is some evidence that suggests there may be a genetic component to the development of intracranial aneurysms, it is not  fully understood. |

|  |  | Having a first-degree relative (such as a parent or sibling) with an intracranial aneurysm may increase your risk of developing one, but it does not necessarily mean that you will develop one or that you will pass it on to your children.  It's important to remember that many factors can contribute to the development of intracranial aneurysms, including high blood pressure, smoking, and atherosclerosis. It's important to manage any risk factors that you may have and to seek medical attention if you experience any symptoms of an intracranial aneurysm, such as a  sudden severe headache or neurological symptoms. |
| --- | --- | --- |
| ChatGPT 4.0 | Re 1 | Intracranial aneurysms can have genetic components, but the majority of cases are not directly inherited. Most intracranial aneurysms are considered sporadic, meaning they occur without a clear genetic cause. However, certain genetic conditions and family history can increase the risk of developing an intracranial aneurysm.  A small percentage of intracranial aneurysms are associated with hereditary conditions, such as:  Autosomal dominant polycystic kidney disease (ADPKD): A genetic disorder that primarily affects the kidneys, but is also associated with an increased risk of intracranial aneurysms.  Ehlers-Danlos syndrome: A group of inherited connective tissue disorders that can affect the blood vessels and increase the risk of aneurysms.  Marfan syndrome: A genetic disorder that affects connective tissue and can weaken the walls of blood vessels, including those in the brain.  Fibromuscular dysplasia: A vascular disease that can cause narrowing and weakening of the arterial walls, increasing the risk of aneurysms.  Having a family history of intracranial aneurysms can also increase the risk for your children. If you have a first-degree relative (parent, sibling, or child) with an intracranial aneurysm, the risk of developing an aneurysm is higher compared to the general population.  It's essential to discuss your medical history and any concerns about the heritability of intracranial aneurysms with your healthcare provider. They can help determine if your children are at an increased risk and provide guidance on any necessary screening or preventive  measures. |

|  |  | Keep in mind that while there may be an increased risk, it does not necessarily mean that your children will develop an intracranial aneurysm. Maintaining a healthy lifestyle, managing risk factors like high blood pressure, and monitoring for any concerning symptoms can help reduce the risk of aneurysm development and related  complications. |
| --- | --- | --- |
|  |  | While I am not a doctor, I can provide you with some general information about intracranial aneurysms and genetic risk. Most intracranial aneurysms are sporadic, meaning they occur randomly and are not directly inherited. However, there can be a genetic predisposition to developing aneurysms in some cases, particularly if you have a family history of aneurysms or certain genetic conditions. |
|  | Re 2 | Familial aneurysms, where multiple family members have intracranial aneurysms, are relatively rare. They are more likely to occur in families with specific genetic disorders, such as polycystic kidney disease, Marfan syndrome, Ehlers-Danlos syndrome, or other connective tissue disorders. |
|  |  | While the overall risk of passing an intracranial aneurysm to your children is relatively low, having a family history of aneurysms or a genetic condition that increases the risk of aneurysms may increase their risk. It is important to discuss your concerns with a healthcare provider or genetic counselor. They can provide you with personalized information about your children's risk and recommend  appropriate screening or preventive measures if necessary. |
|  |  | Intracranial aneurysms can have a genetic component, meaning that there may be a slightly increased risk for your children if you have an intracranial aneurysm. However, the overall hereditary risk is generally considered to be low. Most intracranial aneurysms are sporadic, which means they occur without a clear genetic link. |
|  | Re 3 | Certain genetic conditions or family histories may increase the risk of intracranial aneurysms, such as: |
|  |  | Autosomal dominant polycystic kidney disease (ADPKD) Ehlers-Danlos syndrome (vascular type)  Marfan syndrome  Familial intracranial aneurysms (a rare condition where multiple family members are affected by aneurysms)  It's important to discuss your family history and any concerns you  have about passing on an intracranial aneurysm to your children with |

|  |  | your healthcare provider. They can provide you with personalized advice based on your specific situation and, if necessary, recommend genetic counseling or testing to further assess your family's risk.  In general, maintaining a healthy lifestyle, managing blood pressure, and avoiding smoking can help reduce the risk of intracranial aneurysms and other cerebrovascular conditions. |
| --- | --- | --- |

| Question 11 | | What should I do if my blood pressure is still high after taking  amlodipine? |
| --- | --- | --- |
|  |  | If your blood pressure remains high even after taking amlodipine, there are several steps you can take: |
|  |  | Consult your doctor: It is important to let your doctor know that your blood pressure is still high despite taking medication. Your doctor may adjust your dosage, prescribe a different medication, or suggest other lifestyle changes that may help lower your blood pressure. |
|  |  | Monitor your blood pressure regularly: It is recommended to monitor your blood pressure at home regularly and keep a record to share with your doctor. This can help your doctor determine whether your medication is working effectively. |
|  | Re 1 | Make lifestyle changes: Along with taking medication, lifestyle changes can also help lower blood pressure. This includes eating a healthy diet, reducing salt intake, increasing physical activity, maintaining a healthy weight, and avoiding smoking and excessive alcohol consumption. |
| ChatGPT 3.5 |  | Consider other medications: If amlodipine is not effective in lowering your blood pressure, your doctor may suggest adding another medication or switching to a different medication. There are several classes of medications that can help lower blood pressure, including diuretics, ACE inhibitors, and beta-blockers. |
|  |  | It is important to follow your doctor's advice and take medications as prescribed. Do not stop taking your medication without consulting your doctor, as this can be dangerous and can cause a sudden increase  in blood pressure. |
|  |  | If your blood pressure remains high after taking amlodipine, it is important to talk to your healthcare provider. They may recommend adjusting your medication dosage, switching to a different medication, or making other changes to your treatment plan. |
|  | Re 2 | In some cases, lifestyle changes such as increasing physical activity, reducing sodium intake, maintaining a healthy weight, limiting alcohol consumption, and managing stress can also help lower blood pressure. |
|  |  | It is important to continue taking your medication as prescribed by your healthcare provider, even if your blood pressure is still high. Stopping or changing your medication without medical supervision |

|  |  | can be dangerous and may lead to serious complications.  If you experience any side effects from your medication or have concerns about its effectiveness, talk to your healthcare provider. They can provide guidance on how to safely and effectively manage  your blood pressure. |
| --- | --- | --- |
|  | Re 3 | If your blood pressure is still high after taking amlodipine, there are several things you can do:  Consult your healthcare professional: It's important to let your healthcare professional know if your blood pressure is still high despite taking amlodipine as prescribed. They may adjust your dose or switch you to a different medication.  Make lifestyle changes: Lifestyle changes such as eating a healthy diet, exercising regularly, maintaining a healthy weight, quitting smoking, and limiting alcohol intake can all help to lower blood pressure. Even small changes can make a difference.  Monitor your blood pressure: Keep track of your blood pressure at home using a blood pressure monitor. This can help you and your healthcare professional to track your progress and make any necessary adjustments to your treatment plan.  Consider combination therapy: In some cases, a combination of medications may be needed to lower blood pressure. Your healthcare professional may recommend adding another medication to your treatment plan, such as a diuretic or ACE inhibitor.  It's important to follow your healthcare professional's advice and not to make any changes to your medication or treatment plan without their guidance. Uncontrolled high blood pressure can lead to serious health problems, so it's important to take steps to lower your blood  pressure if it's still high after taking amlodipine. |
| ChatGPT 4.0 | Re 1 | I am not a doctor, but I can provide you with some general advice regarding high blood pressure management. If your blood pressure remains high after taking amlodipine, it's important to discuss this with your healthcare provider. They may recommend one or more of the following:  Adjust the dosage: Your doctor may decide to adjust the dosage of amlodipine or the frequency at which you take it.  Add another medication: In some cases, your doctor may recommend |

|  |  | adding another blood pressure-lowering medication to your treatment plan. This is known as combination therapy and can include medications from different classes, such as diuretics, beta- blockers, ACE inhibitors, or angiotensin II receptor blockers (ARBs). |
| --- | --- | --- |
|  |  | Review your current medications: Some medications can interfere with the effectiveness of blood pressure-lowering drugs or even raise blood pressure. Your healthcare provider may review your current medications and make necessary adjustments. |
|  |  | Evaluate lifestyle factors: Ensuring that you are following a heart- healthy lifestyle is crucial for blood pressure management. This includes maintaining a healthy diet (such as the DASH diet), engaging in regular physical activity, managing stress, maintaining a healthy weight, limiting alcohol consumption, and quitting smoking. |
|  |  | Monitor your blood pressure: Regularly monitoring your blood pressure at home and keeping track of your readings can provide valuable information for your healthcare provider to assess the effectiveness of your treatment plan. |
|  |  | Rule out secondary hypertension: In some cases, high blood pressure may be caused by an underlying medical condition, such as kidney disease or hormonal imbalances. Your doctor may perform additional tests to identify any potential underlying causes. |
|  |  | Remember that managing high blood pressure often requires ongoing adjustments to find the most effective treatment plan. It's crucial to maintain regular communication with your healthcare provider and follow their recommendations. Never make changes to your  medications or treatment plan without consulting your doctor. |
|  | Re 2 | I am not a doctor, but I can provide you with some general information about managing high blood pressure. If your blood pressure remains high after taking amlodipine or any other medication, it is essential to consult with your healthcare provider. They will be able to assess your situation and may adjust your medication, dosage, or add additional medications to help manage your blood pressure. |
|  |  | In addition to medication, there are some lifestyle changes you can implement to help lower your blood pressure: |
|  |  | Maintain a healthy diet: Adopt a heart-healthy diet that includes fruits, vegetables, whole grains, lean proteins, and low-fat dairy, |

|  |  | while limiting saturated and trans fats, sodium, and added sugars.  Exercise regularly: Aim for at least 150 minutes of moderate- intensity aerobic activity or 75 minutes of vigorous-intensity aerobic activity per week to help lower blood pressure and improve overall cardiovascular health.  Maintain a healthy weight: Losing excess weight can help lower blood pressure.  Manage stress: Practice stress reduction techniques such as deep breathing, meditation, yoga, or engaging in hobbies and activities you enjoy.  Limit alcohol consumption: If you drink alcohol, do so in moderation—up to one drink per day for women and up to two drinks per day for men.  Don't smoke: If you smoke, quitting is one of the best things you can do for your heart health and overall well-being.  Monitor your blood pressure: Keep track of your blood pressure regularly to ensure that it remains within a healthy range.  Always consult with your healthcare provider before making any changes to your medication or implementing lifestyle changes, especially if you have a pre-existing medical condition. They can provide personalized advice tailored to your specific needs and  circumstances. |
| --- | --- | --- |
|  | Re 3 | I'm not a doctor, but I can offer you some general advice. If your blood pressure remains high after taking amlodipine or any other prescribed medication, it's important to consult with your healthcare provider. They may need to adjust your medication dosage, change medications, or add additional medications to help manage your blood pressure more effectively.  In addition to taking your prescribed medications, consider the following lifestyle modifications to help lower blood pressure:  Maintain a healthy diet: Consume a balanced diet rich in fruits, vegetables, whole grains, lean proteins, and low-fat dairy products. Limit your intake of sodium, saturated and trans fats, and added sugars. The DASH (Dietary Approaches to Stop Hypertension) diet is specifically designed to help lower blood pressure.  Exercise regularly: Aim for at least 150 minutes of moderate- intensity aerobic exercise or 75 minutes of vigorous-intensity aerobic exercise per week, spread across multiple days.  Maintain a healthy weight: Losing excess weight can help lower blood pressure. Even a modest weight loss can have a significant |

|  |  | impact on blood pressure.  Limit alcohol consumption: If you drink alcohol, do so in moderation. Excessive alcohol consumption can raise blood pressure.  Don't smoke: Smoking can damage blood vessels and increase the risk of high blood pressure. Quitting smoking can improve your overall cardiovascular health.  Manage stress: Engage in stress reduction techniques such as deep breathing, meditation, yoga, or participating in hobbies and activities that you enjoy.  Monitor your blood pressure: Regularly check your blood pressure to ensure it remains within a healthy range. Share your blood pressure readings with your healthcare provider to help them make informed decisions about your treatment.  Remember, always consult with your healthcare provider before making any significant changes to your lifestyle or medication regimen, especially if you have a pre-existing medical condition or concerns about your blood pressure. |
| --- | --- | --- |

| Question 12 | | Do my emotions have impact on my brain vessels? |
| --- | --- | --- |
|  |  | Yes, your emotions can have an impact on your brain vessels. Emotions can cause changes in the body's physiology, including changes in blood pressure, heart rate, and hormonal levels, which can affect the blood vessels in the brain. |
|  |  | For example, when you experience stress, your body releases hormones such as adrenaline and cortisol, which can increase blood pressure and cause blood vessels to constrict. This can restrict blood flow to the brain and increase the risk of developing a stroke or other cerebrovascular diseases. |
|  | Re 1 | On the other hand, positive emotions such as happiness and contentment can have a beneficial effect on the brain vessels. Studies have shown that positive emotions can lead to the release of endorphins and other neurotransmitters that help relax blood vessels, improve blood flow, and reduce inflammation. |
| ChatGPT 3.5 |  | Chronic stress and negative emotions can also contribute to the development of conditions such as anxiety and depression, which have been linked to increased risk of cardiovascular disease and stroke.  Therefore, it is important to pay attention to your emotional well- being and manage stress effectively to maintain good brain health and reduce the risk of cerebrovascular diseases. |
|  |  | Yes, emotions can have an impact on brain vessels. Stressful or negative emotions such as anger, fear, or anxiety can cause the release of stress hormones such as cortisol and adrenaline. These hormones can increase heart rate and blood pressure, which can affect blood vessels throughout the body, including in the brain. |
|  | Re 2 | Chronic stress and negative emotions have been linked to a higher risk of developing cardiovascular diseases, including cerebrovascular diseases such as stroke. Chronic stress can also cause inflammation, which can contribute to the development of atherosclerosis (hardening and narrowing of the arteries) and increase the risk of stroke. |
|  |  | On the other hand, positive emotions such as happiness and love have been shown to have a protective effect on the cardiovascular system, including the brain vessels. Positive emotions can lead to the release of endorphins, which can have a relaxing effect on blood vessels and  help reduce stress. |

|  |  | Therefore, it is important to manage stress and negative emotions through techniques such as relaxation, meditation, exercise, and social support. These techniques can help reduce the impact of negative emotions on the brain vessels and improve overall  cardiovascular health. |
| --- | --- | --- |
|  | Re 3 | Yes, emotions can have an impact on brain vessels. When we experience strong emotions such as anger, fear, or excitement, our bodies release stress hormones like adrenaline and cortisol, which can cause our blood vessels to constrict and increase blood pressure. This response is known as the "fight or flight" response, and it evolved as a way to prepare our bodies to respond to a perceived threat or danger.  However, when this response is activated repeatedly or for prolonged periods of time, it can lead to chronic high blood pressure, which can damage blood vessels and increase the risk of stroke and other cardiovascular diseases.  In addition, studies have also shown that positive emotions like happiness, joy, and gratitude can have a protective effect on the cardiovascular system by reducing stress hormones and promoting relaxation. This is why it's important to find healthy ways to manage and regulate our emotions, such as through mindfulness, meditation, exercise, and social support. |
| ChatGPT 4.0 | Re 1 | Emotions can indeed have an impact on your brain vessels and overall cardiovascular system. When you experience strong emotions, such as stress, anxiety, anger, or fear, your body releases stress hormones like adrenaline and cortisol. These hormones can cause an increase in heart rate and blood pressure, and they can temporarily narrow your blood vessels.  Although these changes are part of the body's natural stress response and are usually short-lived, chronic exposure to stress and strong negative emotions can have long-term effects on your cardiovascular system. Persistent stress and emotional strain can contribute to consistently elevated blood pressure (hypertension), increased inflammation, and damage to blood vessels over time. This may increase the risk of developing cardiovascular issues, such as atherosclerosis, heart attacks, and strokes.  It's essential to manage stress and emotional well-being as part of a heart-healthy lifestyle. Some strategies to help cope with stress and promote emotional health include: |

|  |  | Regular physical activity: Exercise has been shown to reduce stress, improve mood, and support overall cardiovascular health.  Relaxation techniques: Practicing relaxation techniques, such as deep breathing, meditation, progressive muscle relaxation, or guided imagery, can help reduce stress and promote emotional well-being. Social support: Maintaining strong social connections and seeking support from friends, family, or support groups can help you better manage stress and emotional challenges.  Cognitive-behavioral therapy (CBT): CBT is a form of psychotherapy that can help you identify and change negative thought patterns and develop healthier coping strategies for managing stress and emotions.  Adequate sleep: Prioritizing good sleep hygiene and getting sufficient, restful sleep can help reduce stress and support overall health.  Balanced diet: Eating a balanced diet rich in fruits, vegetables, whole grains, lean proteins, and healthy fats can help support mood and overall health.  Limiting alcohol and caffeine: Reducing intake of alcohol and caffeine, which can exacerbate stress and anxiety, can help promote emotional well-being.  Time management: Developing effective time management and organization skills can help reduce stress and improve work-life balance.  If you're struggling with managing stress or emotions, consider seeking support from a mental health professional, such as a psychologist, psychiatrist, or therapist, to help develop personalized  strategies for coping and improving your emotional well-being. |
| --- | --- | --- |
|  | Re 2 | Emotions can have an impact on various aspects of your health, including your brain vessels and cardiovascular system. Stress, in particular, has been shown to affect blood vessels and blood pressure. When you experience stress or strong emotions, your body releases stress hormones like cortisol and adrenaline, which can cause an increase in heart rate and blood pressure.  Over time, chronic stress or persistently high levels of stress hormones can lead to inflammation and damage to the blood vessels, including those in the brain. This damage can contribute to the development of atherosclerosis (hardening and narrowing of the arteries), which in turn can increase the risk of stroke and other cerebrovascular events.  Managing stress and maintaining good emotional health is essential |

|  |  | for overall well-being and cardiovascular health. Here are some strategies that can help: |
| --- | --- | --- |
|  |  | Practice relaxation techniques: Deep breathing exercises, meditation, progressive muscle relaxation, and mindfulness can help reduce stress and promote relaxation.  Engage in physical activity: Regular exercise can help alleviate stress, improve mood, and promote cardiovascular health.  Get adequate sleep: Aim for 7-9 hours of sleep per night, as sleep is crucial for emotional well-being and overall health.  Maintain a healthy diet: Eating a balanced diet can help support emotional health and reduce the risk of cardiovascular issues.  Seek social support: Connect with friends, family members, or support groups to help you cope with stress and share your feelings. Consider professional help: If stress and emotional challenges become overwhelming, consider speaking with a mental health professional, such as a psychologist, psychiatrist, or counselor.  While emotions can have an impact on brain vessels, it is important to note that multiple factors contribute to cerebrovascular health. Genetics, lifestyle choices, and medical conditions all play a role. It's essential to maintain a healthy lifestyle, manage stress, and follow your healthcare provider's recommendations to minimize the risk of  cerebrovascular issues. |
|  |  | Emotions can have both short-term and long-term effects on your brain vessels and overall cardiovascular health. When you experience intense emotions, such as stress, anxiety, anger, or sadness, your body releases stress hormones like adrenaline and cortisol, which can cause temporary changes in your blood vessels, heart rate, and blood pressure. |
|  | Re 3 | In the short term, these changes are part of your body's natural "fight or flight" response, which helps you react to perceived threats or challenges. However, if you experience chronic stress or negative emotions over an extended period, it can have a more lasting impact on your cardiovascular health. |
|  |  | Chronic stress and negative emotions can contribute to: |
|  |  | Increased blood pressure: Ongoing emotional stress can lead to consistently elevated blood pressure, which is a significant risk factor for both heart disease and stroke. |
|  |  | Atherosclerosis: Chronic stress may contribute to the buildup of plaque in your arteries (atherosclerosis), which can restrict blood |

|  |  | flow and increase the risk of a stroke or heart attack.  Irregular heart rhythms: Intense emotions can sometimes trigger irregular heart rhythms (arrhythmias), which can increase the risk of stroke or other cardiovascular events.  Inflammation: Chronic stress can contribute to inflammation, which is associated with a higher risk of heart disease and stroke.  Unhealthy coping mechanisms: Prolonged stress or negative emotions can lead some people to adopt unhealthy habits, such as overeating, smoking, or excessive alcohol consumption, which can further increase the risk of cardiovascular problems.  It's essential to recognize the connection between your emotional well-being and cardiovascular health and take steps to manage stress and negative emotions effectively. This may include engaging in stress-reduction techniques, such as deep breathing exercises, meditation, yoga, or pursuing hobbies and activities that bring you joy and relaxation. Additionally, seeking support from friends, family, or a mental health professional can be beneficial in managing  stress and maintaining emotional well-being. |
| --- | --- | --- |

| Question 13 | | I had a stroke. Can I drink coffee or tea? |
| --- | --- | --- |
| ChatGPT 3.5 | Re 1 | Whether or not you can drink coffee or tea after a stroke depends on several factors, including the severity and type of stroke, any medications you are taking, and any other medical conditions you may have.  In general, moderate caffeine intake is considered safe for most people, including those who have had a stroke. The American Heart Association recommends that most adults can safely consume up to 400 milligrams of caffeine per day, which is roughly the amount in four cups of brewed coffee. However, it is always best to check with your doctor to determine what is safe and appropriate for your individual situation.  Caffeine can affect blood pressure and heart rate, so if you have high blood pressure or a heart condition, your doctor may advise you to limit your caffeine intake or avoid it altogether. Some medications used to treat stroke and other cardiovascular conditions can also interact with caffeine, so it is important to discuss any concerns with your doctor or pharmacist.  It is also important to note that coffee and tea can have other potential health benefits, including antioxidants and anti-inflammatory properties, which may be beneficial for stroke recovery. However, more research is needed to fully understand these effects.  In summary, if you have had a stroke, it is important to discuss your caffeine intake with your doctor, who can provide guidance on what is safe and appropriate for your individual situation. |
|  | Re 2 | If you have had a stroke, it is important to talk to your healthcare provider about whether or not it is safe for you to consume coffee or tea.  Caffeine, which is found in coffee, tea, and many other beverages and foods, can affect blood pressure and heart rate. In some people, caffeine consumption can trigger or worsen high blood pressure, which is a risk factor for stroke.  In general, moderate caffeine consumption (up to 400mg per day, or about 4 cups of coffee) is considered safe for most healthy adults. However, the impact of caffeine on blood pressure and heart rate can vary depending on individual factors such as age, sex, weight, and medical history. |

|  |  | Your healthcare provider can advise you on whether or not it is safe for you to consume caffeine after a stroke, taking into account your individual circumstances and medical history. They may recommend limiting or avoiding caffeine if you have high blood pressure or other  medical conditions that could be worsened by caffeine consumption. |
| --- | --- | --- |
|  | Re 3 | After a stroke, it is generally safe to consume moderate amounts of caffeine in the form of coffee or tea, but it's important to consult with your healthcare professional for personalized advice.  Caffeine can have both positive and negative effects on the body. On the positive side, it can improve alertness, concentration, and cognitive function, which may be beneficial for stroke recovery. It may also have a protective effect against stroke by reducing inflammation and improving blood flow.  On the negative side, caffeine can also increase blood pressure and heart rate, which can be problematic for people who have had a stroke, as high blood pressure is a risk factor for stroke. In addition, caffeine can interfere with sleep, which is important for stroke recovery.  Your healthcare professional will be able to give you specific recommendations based on your individual health status and any medications you may be taking. In general, they may advise you to limit your caffeine intake to no more than 300-400 milligrams per day (equivalent to 2-3 cups of coffee), and to avoid consuming  caffeine later in the day, as this can interfere with sleep. |
| ChatGPT 4.0 | Re 1 | I'm not a doctor, but I can provide you with some general information on this topic. After a stroke, it's essential to discuss your dietary choices, including coffee and tea consumption, with your healthcare provider. They can provide personalized recommendations based on your specific health situation and recovery progress.  In general, moderate consumption of coffee and tea is not considered harmful for most people, and both beverages have been associated with some health benefits. However, it's important to note that both coffee and tea contain caffeine, a stimulant that can temporarily increase heart rate and blood pressure. For some individuals, particularly those with high blood pressure or other cardiovascular risk factors, excessive caffeine intake might not be recommended.  If your healthcare provider approves, you may be able to consume coffee and tea in moderation. Moderate caffeine intake is typically considered to be around 200-300 milligrams per day, which is |

|  |  | roughly equivalent to 2-3 cups of coffee or 4-5 cups of tea. However, your healthcare provider may suggest a different amount based on your individual needs and circumstances.  Additionally, be mindful of added sugars, cream, and other high- calorie additives in coffee and tea, as they can contribute to weight gain and negatively impact overall health. Opt for healthier alternatives such as a small amount of low-fat milk or a sugar substitute if needed.  Always consult your healthcare provider before making changes to your diet or lifestyle, especially after a stroke. They can provide the most accurate and personalized advice for your specific situation. |
| --- | --- | --- |
|  | Re 2 | I'm not a doctor, but I can provide you with some general information on this topic. Moderate consumption of coffee or tea is usually considered safe for most people, even after a stroke, as long as it doesn't interfere with any prescribed medications or medical advice from your healthcare provider. It's essential to consult with your doctor or healthcare professional for personalized advice based on your specific situation and medical history.  Caffeine, which is found in coffee and tea, can have both positive and negative effects on health. Some studies suggest that moderate caffeine consumption may have potential health benefits, such as improving cognitive function and reducing the risk of certain diseases. However, excessive caffeine intake can lead to increased heart rate, elevated blood pressure, and other adverse effects.  If you've had a stroke and would like to consume coffee or tea, consider the following general guidelines:  Limit your intake: Moderate caffeine consumption is typically defined as around 200-300 mg per day, which is approximately 2-4 cups of coffee or 4-6 cups of tea. Keep in mind that individual tolerances to caffeine vary.  Monitor your blood pressure: If you have high blood pressure, it's important to monitor your blood pressure regularly and ensure that caffeine consumption doesn't cause it to rise further.  Consult your healthcare provider: Talk to your doctor about your caffeine consumption and whether it is appropriate for you based on your specific medical history and conditions. |

|  |  | Choose decaffeinated options: If you're concerned about caffeine's effects on your health, consider switching to decaffeinated coffee or tea.  Remember, it's essential to consult with your healthcare provider about your specific situation and follow their recommendations regarding caffeine consumption and other lifestyle choices after a stroke. |
| --- | --- | --- |
|  | Re 3 | I'm not a doctor, but I can provide you with some general information. The effects of coffee and tea on stroke survivors may depend on the individual and the specific circumstances of their stroke. It's important to consult with your healthcare provider before making any dietary changes, including consuming coffee or tea, as they can provide personalized recommendations based on your medical history and current health status.  In general, moderate coffee or tea consumption may not be harmful for most people, and some studies suggest that these beverages might even have some protective effects against stroke when consumed in moderation. However, it's essential to consider the following factors:  Caffeine: Both coffee and tea contain caffeine, which can raise blood pressure and heart rate temporarily. For some individuals, especially those with high blood pressure or a history of heart problems, excessive caffeine intake may not be advisable.  Sugar and calories: Adding large amounts of sugar, cream, or other high-calorie ingredients to your coffee or tea can contribute to weight gain and increased blood sugar levels, which could negatively impact your stroke recovery and overall health.  Interactions with medications: Some medications, including blood thinners or blood pressure medications, may interact with caffeine or other compounds found in coffee and tea. Consult your healthcare provider or pharmacist to ensure there are no potential interactions with your current medications.  Individual tolerability: Some people may be more sensitive to caffeine than others. If you notice that coffee or tea consumption triggers any unwanted symptoms or affects your blood pressure, it's important to discuss this with your healthcare provider.  If your healthcare provider approves, you may be able to enjoy coffee or tea in moderation as part of a balanced and healthy diet. Make sure |

|  |  | to monitor your caffeine intake and pay attention to any potential side  effects or changes in your overall health. |
| --- | --- | --- |
